# Small Extracellular Vesicle‐Derived vWF Induces a Positive Feedback Loop between Tumor and Endothelial Cells to Promote Angiogenesis and Metastasis in Hepatocellular Carcinoma

**DOI:** 10.1002/advs.202302677

**Published:** 2023-06-30

**Authors:** Samuel Wan Ki Wong, Sze Keong Tey, Xiaowen Mao, Hiu Ling Fung, Zhi‐Jie Xiao, Danny Ka Ho Wong, Lung‐Yi Mak, Man‐Fung Yuen, Irene Oi‐Lin Ng, Jing Ping Yun, Yi Gao, Judy Wai Ping Yam

**Affiliations:** ^1^ Department of Pathology School of Clinical Medicine Li Ka Shing Faculty of Medicine The University of Hong Kong Hong Kong; ^2^ Department of Surgery School of Clinical Medicine Li Ka Shing Faculty of Medicine The University of Hong Kong Hong Kong; ^3^ State Key Laboratory of Liver Research, The University of Hong Kong Hong Kong; ^4^ Research Centre The Seventh Affiliated Hospital Sun Yat‐sen University 518107 Shenzhen P. R. China; ^5^ Department of Medicine School of Clinical Medicine Li Ka Shing Faculty of Medicine The University of Hong Kong Hong Kong; ^6^ Department of Pathology Sun Yat‐sen University Cancer Center Guangzhou Guangdong 510060 P. R. China; ^7^ Department of Hepatobiliary Surgery II ZhuJiang Hospital Southern Medical University Guangzhou Guangdong 510280 P. R. China

**Keywords:** feedback signaling, hepatocellular carcinoma, intercellular communication, small extracellular vesicles, von Willebrand factor

## Abstract

Hepatocellular carcinoma (HCC) is a hypervascular malignancy by which its growth and dissemination are largely driven by the modulation of tumor‐derived small extracellular vesicles (sEVs). Proteomic profiling of circulating sEVs of control individuals and HCC patients identifies von Willibrand factor (vWF) to be upregulated progressively along HCC stages. Elevated sEV–vWF levels are found in a larger cohort of HCC–sEV samples and metastatic HCC cell lines compared to their respective normal counterparts. Circulating sEVs of late‐stage HCC patients markedly augment angiogenesis, tumor–endothelial adhesion, pulmonary vascular leakiness, and metastasis, which are significantly compromised by anti‐vWF antibody. The role of vWF is further corroborated by the enhanced promoting effect of sEVs collected from vWF‐overexpressing cells. sEV–vWF modulates endothelial cells through an elevated level of vascular endothelial growth factor A (VEGF‐A) and fibroblast growth factor 2 (FGF2). Mechanistically, secreted FGF2 elicits a positive feedback response in HCC via the FGFR4/ERK1 signaling pathway. The co‐administration of anti‐vWF antibody or FGFR inhibitor significantly improves the treatment outcome of sorafenib in a patient‐derived xenograft mouse model. This study reveals mutual stimulation between HCC and endothelial cells by tumor‐derived sEVs and endothelial angiogenic factors, facilitating angiogenesis and metastasis. It also provides insights into a new therapeutic strategy involving blocking tumor–endothelial intercellular communication.

## Graphical abstract

Tumor small extracellular vesicle (sEV)‐derived von Willibrand factor (vWF) induces a positive feedback loop between tumor and endothelial cells to promote angiogenesis and metastasis in hepatocellular carcinoma (HCC). This study provides insights into the exploitation of blocking sEV‐mediated tumor‐endothelial communication as a therapeutic strategy for HCC patients.

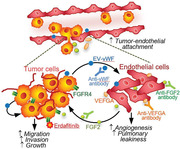

## 1 Introduction

As the sixth most common cancer worldwide, hepatocellular carcinoma (HCC) accounts for an estimated 75–85% of primary liver cancer cases.^[^ ^1^ ^]^ HCC is commonly diagnosed at an advanced stage with extrahepatic metastasis, at which point curative treatment is limited. With the limited availability of therapeutic options, HCC is the fourth leading cause of cancer death.^[^ ^2^ ^]^ HCC is a hypervascular tumor with an extensive architecture of blood vessels that facilitates the dissemination of cancer cells through the hematogenous route. Neoangiogenesis is defined as the formation of a new vascular network, which is necessary to supply adequate oxygen and nutrients to cancer cells for their survival and growth.^[^ ^3^ ^]^ Enhanced vascularization is also essential for cancer metastasis, providing an exit route for tumor cells to enter the blood circulation such that they can disseminate to distant loci and establish secondary metastatic tumors in a conducive microenvironment.^[^ ^4^ ^]^

Small extracellular vesicles (sEVs) are membranous vesicles secreted by cells, typically ranging from 30 to 150 nm, which play vital roles in cellular intercommunication.^[^ ^5^ ^]^ The transfer of their embedded functional cargos (including oncoproteins, RNA species, and lipids) allows sEVs to modulate the versatile signaling and phenotypes of the recipient cells.^[^ ^6^ ^]^ Accumulating evidence suggests that tumor‐derived sEVs, also named exosomes, are capable of affecting angiogenic signaling in multiple cancers. Vascular cells are indispensable to tumor angiogenesis, which is crucial for cancer survival and metastasis. It has been reported that Gas6, which was first identified in tumor perivascular cell‐derived sEVs, recruits endothelial progenitor cells for tumor revascularization by the Axl pathway in colorectal cancer.^[^ ^7^ ^]^ CAT1 is another potential sEV biomarker of colorectal cancer that regulates vascular endothelial cells through arginine‐associated metabolic and ERK/p38 pathways.^[^ ^8^ ^]^ Glioblastoma cell‐secreted sEVs carry a significantly high level of vascular endothelial growth factor A (VEGF‐A), which directly acts on human brain endothelial cells to promote angiogenesis.^[^ ^9^ ^]^ The angiopoietin‐2 pathway activated by sEVs derived from gastric cancer cells promotes angiogenesis through enhanced endothelial migration, invasion, and tube formation.^[^ ^10^ ^]^ In HCC, exosomal miR‐210 modulates the interaction between tumor and vascular cells by inhibiting endothelial SMAD4 and STAT6.^[^ ^11^ ^]^ Due to the hypervascular nature of HCC, the involvement of tumor‐derived sEVs in HCC angiogenesis warrants in‐depth investigation.

In our previous proteomic profiling of sEVs, von Willibrand factor (vWF) was found to be significantly upregulated in the circulating sEVs of HCC patients compared to control individuals.^[^ ^12^ ^]^ vWF is a multimeric protein synthesized by endothelial cells, platelets, and sub‐endothelial connective tissues. It is important in platelet adhesion at the site of vascular injury and in the formation of blood vessels. It has been suggested that the expression of vWF in tumors is an indicator of activated endothelium or angiogenesis.^[^ ^13^ ^]^ An elevated level of plasma vWF is found in patients with various cancers.^[^ ^14^ ^]^ In a recent study, profiling a large cohort of sEVs obtained from patients with different cancer types, sEV–vWF together with other immunoglobin‐related proteins was found to be predictive for detecting cancers.^[^ ^15^ ^]^ Despite an association of an enhanced sEV–vWF level with various types of human cancers, the functional role and mechanistic basis of vWF in the form of EVs have never been extensively characterized.

In this study, we demonstrate that mutual stimulation between HCC and endothelial cells mediated by tumor cell‐derived sEV–vWF and angiogenic factors released by endothelial cells resulted in an enhancement of angiogenesis and metastasis. We also revealed the clinical relevance of sEV–vWF in HCC development and the potential of its application in liquid biopsies for the diagnosis of HCC. Last, our findings implicate the blockade of communication between cancer and endothelial cells as an effective therapeutic option for HCC patients.

## 2 Results

### 2.1 HCC Patient‐Derived sEVs Promote Angiogenesis

**Figure 1:**
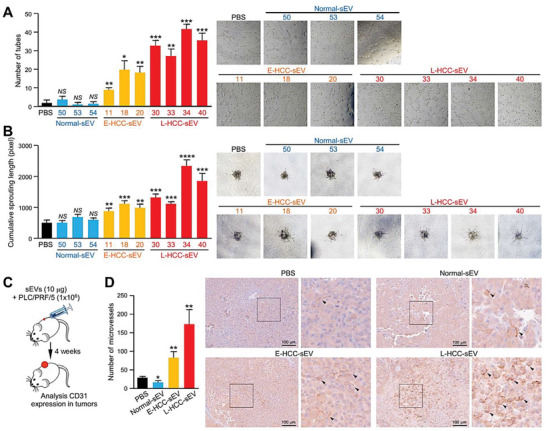
Circulating sEVs from HCC patients promote angiogenesis in vitro and in vivo. A) Tube formation assay of HUVECs pretreated with PBS, circulating sEVs of control individuals (normal) (*n* = 3), and HCC patients at the early stage (E‐HCC) (*n* = 3) and late stage (L‐HCC) (*n* = 4). B) HUVECs pretreated with the indicated sEVs were subjected to an endothelial sprouting assay. C) Schematic diagram of the in vivo Matrigel assay. PLC/PRF/5 cells were subcutaneously co‐injected with PBS, normal, E‐HCC, or L‐HCC‐sEVs (*n* = 5 or 15 mice in total). D) Immunohistochemistry of excised tumors with CD31 staining indicating microvessel formation. Quantification of the microvessels is shown. Representative images of CD31 staining and an enlarged image of the region in the inset box are shown. Scale bar: 100 µm. Data are presented as the mean ± SEM. *P < 0.05, **P < 0.01; ***P < 0.001. *P* < 0.05 was regarded as statistically significant. NS, not significant.

A previous study demonstrated that circulating sEVs of patients with early HCC (E‐HCC‐sEVs) and late HCC (L‐HCC‐sEVs) but not control individuals (normal‐sEVs) promote cancer stemness, motility, tumorigenesis, and metastasis.^[^ ^12^ ^]^ HCC is a hypervascular malignancy in which the extensive architecture of blood vessels facilitates the dissemination of cancer cells through the hematogenous route; we questioned whether HCC‐sEVs modulate endothelial cells to facilitate vasculature development, tumorigenesis, and metastasis. Intriguingly, both E‐HCC‐sEVs and L‐HCC‐sEVs enhanced the tube formation and endothelial sprouting ability of Human umbilical vein endothelial cell (HUVEC), while the latter exhibited a greater promoting effect on angiogenesis (**Figure** **1**A,B). Such a promoting capacity was not observed for normal‐sEVs. Our previous study reported the augmenting effect of L‐HCC‐sEVs in liver tumor formation and distant metastasis.^[^ ^12^ ^]^ Here, we observed that more extensive microvessels were formed in tumors derived from subcutaneous co‐injection of PLC/PRF/5 cells with HCC‐sEVs than in tumors developed from cells co‐injected with or without normal‐sEVs (Figure 1C,D). These findings revealed the ability of HCC‐patient‐derived sEVs to induce angiogenic vascularization.

### 2.2 Elevation of vWF Is Found in Circulating sEVs of HCC Patients

**Figure 2:**
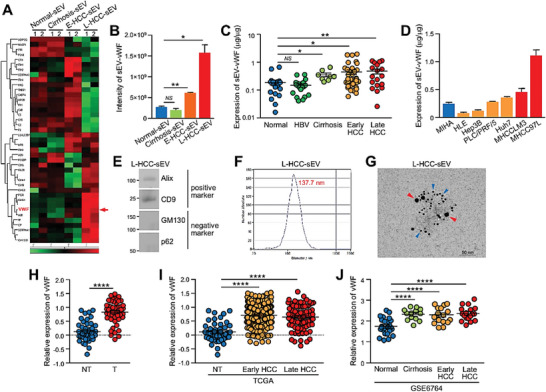
High level of vWF in circulating sEVs of HCC patients. A) Proteins were extracted from sEVs of control individuals (normal‐sEVs), patients with cirrhosis (cirrhosis‐sEVs), early HCC (E‐HCC‐sEVs), and late HCC (L‐HCC‐sEVs), and subjected to proteomic profiling using mass spectrometry (technical duplicate/sample). Heat map reveals the proteomic profiles of sEV proteins that were expressed in all sEVs tested and with *P* < 0.5. B) The level of vWF in the indicated sEVs detected by mass spectrometry. C) The level of vWF in the circulating sEVs of non‐HCC individuals (control) (*n* = 18), HBV patients (*n* = 20), patients with cirrhosis (*n* = 8), early HCC (*n* = 37), and late HCC (*n* = 18) was analyzed by ELISA. D) Level of vWF in sEVs collected from the conditioned medium (CM) of the immortalized normal liver cell line MIHA, nonmetastatic HCC cell lines (HLE, Hep3B, PLC/PRF/5, and Huh7) and metastatic HCC cell lines (MHCCLM3 and MHCC97 L) was determined by ELISA (*n* = 3). E) Western blotting of positive and negative sEV markers in circulating sEVs obtained from the serum of late‐stage HCC patients (L‐HCC‐sEVs). F) Size distribution of L‐HCC‐sEVs measured by ZetaView TWIN‐NTA PMX‐220. G) Representative electron microscopic images of the L‐HCC‐sEVs co‐stained by anti‐CD63 and anti‐vWF antibodies with gold‐conjugated secondary antibody. Red arrowhead indicates vWF and blue arrowhead indicates CD63. Scale bar, 50 nm. H) The vWF mRNA expression in 50 cases of paired tumor and nontumorous (NT) liver tissues by qPCR. I) Expression of vWF in the TCGA database of liver cancer with 50 nontumorous (NT), 255 early‐HCC and 90 late‐HCC cases. J) Expression of vWF in the GSE6764 database of liver cancer with 27 normal, 13 cirrhosis, 17 early HCC, and 18 late HCC cases. Data are presented as the mean ± SEM. *P < 0.05, **P < 0.01; and ****P < 0.0001. *P* < 0.05 was regarded as statistically significant.

To comprehensively ascertain the differential biological activities between the sEVs of healthy controls and patients with cirrhosis and HCC, our previous proteomic profiling of normal‐sEVs, circulating sEVs of patients with cirrhosis (cirrhosis‐sEVs), E‐HCC‐sEVs, and L‐HCC‐sEVs identified differentially expressed proteins between control individuals and patients (PRoteomics IDEntifications (PRIDE) database: PXD025522)^[^ ^12^ ^]^ (**Figure** **2**A). Among the top listed differentially expressed proteins (Table S1, Supporting Information), vWF increased progressively during HCC development with fivefold upregulation in L‐HCC‐sEVs compared with normal‐sEVs (Figure 2B). The function of vWF in angiogenesis implicates that vWF might be a potential regulator of circulating sEV of HCC patients in modulating endothelial cells as shown in Figure 1. Our finding was in concordance with the previous identification of sEV–vWF as a potential biomarker of HCC using a data‐independent acquisition method.^[^ ^16^ ^]^ The upregulation of sEV–vWF in HCC patients was further validated in an independent cohort of 101 samples comprising circulating sEVs obtained from control subjects without liver diseases and patients with chronic hepatitis B virus infection, cirrhosis, and HCC at early and advanced stages. sEV–vWF was significantly upregulated in patients with cirrhosis and upregulated even higher in accordance with tumor stage (Figure 2C). The level of vWF in sEVs collected from the conditioned medium (CM) of normal liver and HCC cell lines was found to be well correlated with the metastatic potential of the cell lines (Figure 2D). Using immunogold labeling electron microscopy, vWF was shown to be localized on the surface of sEVs from patients at an advanced tumor stage (Figure 2E–G). In accordance with the elevated protein level of vWF in the sEVs of HCC patients and cell lines, vWF mRNA expression was upregulated in tumor tissue relative to the normal tissue in 50 paired in‐house cases of tumor and nontumorous (NT) liver tissues and in samples from the TCGA and GSE6764 databases of liver cancer (Figure 2H–J). These findings point to the clinical significance of vWF upregulation in HCC.

### 2.3 vWF‐Enriched sEVs Promote Angiogenesis, Endothelial Leakiness, and Tumor–Endothelial Interactions

**Figure 3:**
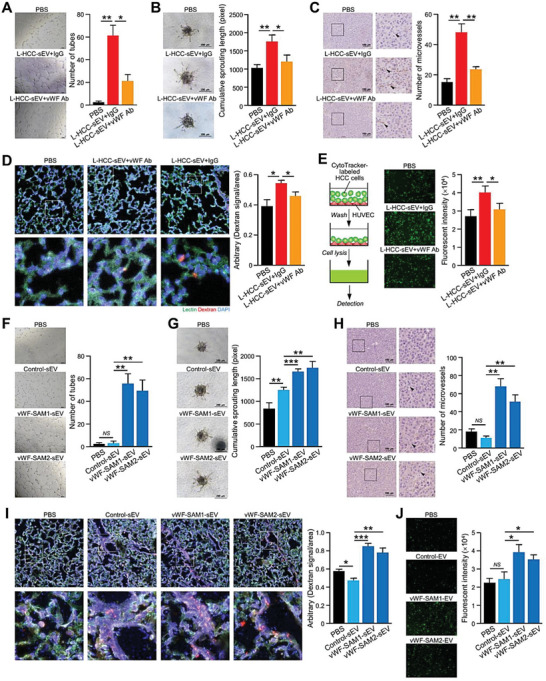
sEV–vWF is involved in in vivo angiogenesis, tumor–endothelial adhesion, and vascular leakiness. A) Tube formation (*n* = 3) and B) endothelial sprouting (*n* = 5) assays were performed using HUVECs treated with L‐HCC‐sEVs in the presence of control IgG or anti‐vWF antibody. The number of tubes and lengths of the sprouts were analyzed. C) In vivo Matrigel plug angiogenesis assay by subcutaneous co‐injection of PLC/PRF/5 cells with the indicated sEVs and antibodies (*n* = 5 or 15 mice in total). Representative images showing immunohistochemical staining of CD31 in the Matrigel plugs. Scale bar: 100 µm. An enlarged image of the region in the inset box is shown. The number of microvessels was quantified. D) Fluorescent images showing vascular permeability of the mouse lungs after intravenous injection of Texas Red‐Dextran, FITC‐Lectin, and L‐HCC‐sEVs together with either control IgG or anti‐vWF antibody (*n* = 3 or 9 mice in total). The Texas Red signal was quantified. E) Tumor–endothelial adhesion assay using CytoTracker‐labeled PLC/PRF/5 cells and L‐HCC‐sEV‐pretreated HUVECs in the presence of control IgG or anti‐vWF antibody (*n* = 3). HUVECs pretreated with control (control‐sEVs) and vWF‐enriched sEVs (vWF‐SAM1‐sEVs and vWF‐SAM2‐sEVs) were subjected to F) tube formation (*n* = 3) and G) endothelial sprouting (*n* = 5) assays. The number of tubes and lengths of the sprouts were counted. H) In vivo Matrigel plug angiogenesis assays by subcutaneous co‐injection of PLC/PRF/5 cells with control‐ and vWF‐SAM‐sEVs (*n* = 5 or 20 mice in total). Representative images showing CD31 expression in the Matrigel plugs. Scale bar: 100 µm. I) Fluorescent images showing vascular permeability of the mouse lungs after intravenous injection of Texas Red‐Dextran, FITC‐Lectin, Control, and vWF‐SAM‐sEVs (*n* = 3 or 12 mice in total). J) HUVECs pretreated with control‐ and vWF‐SAM‐sEVs were subjected to a tumor–endothelial adhesion assay with CytoTracker‐labeled PLC/PRF/5 cells (*n* = 3). Data are presented as the mean ± SEM. *P < 0.05, **P < 0.01; and ***P < 0.001. *P* < 0.05 was regarded as statistically significant. NS, not significant.

Secreted vWF has been reported to regulate blood vessel formation, which is crucial to tumor formation and metastasis.^[^ ^17^ ^]^ Nevertheless, the functional significance of vWF‐enriched sEVs in HCC has not been investigated. The presence of vWF on the surface of sEVs enabled the use of a neutralizing antibody to block its functions. To delineate whether vWF plays a pivotal role in the activating capacity of L‐HCC‐sEVs in angiogenesis, the promoting effect of L‐HCC‐sEVs on angiogenesis was compared in the presence of control and anti‐vWF antibodies. Consistently, the anti‐vWF antibody remarkably inhibited the in vitro tube forming and sprouting capabilities of HUVECs and suppressed in vivo microvessel formation in atrigel plugs derived from PLC/PRF/5 cells induced by L‐HCC‐sEVs (**Figure** **3**A–C).

Tumor–endothelial cell attachment and extravasation of tumor cells from the vasculature are critical to the success of metastasis. We therefore examined whether L‐HCC‐sEVs affected the permeability of blood vessels and the adhesion between tumors and endothelial cells. A pulmonary vascular leakiness assay was performed in mice by intravenous injection of Texas Red‐Dextran, FITC‐Lectin, and L‐HCC‐sEVs together with either control immunoglobulin G (IgG) or anti‐vWF antibody. Dextran was used as an indicator of vessel leakiness, while lectin was used to label the pulmonary vasculature. Compared to mice without sEV injection, injection with L‐HCC‐sEVs resulted in more areas of diffused dextran, indicating enhanced endothelial permeability. This enhancement in dextran intensity was suppressed in mice co‐injected with L‐HCC‐sEVs and anti‐vWF antibody (Figure 3D). The anti‐vWF antibody also significantly reduced the tumor–endothelial attachment between HUVECs and PLC/PRF/5 cells (Figure 3E).

Driven by the observation of the blockade of endothelial cell modulation by L‐HCC‐EVs when using an anti‐vWF antibody, the effect of sEVs overexpressing vWF was investigated. vWF‐enriched sEVs were collected from stable control and vWF‐overexpressing cells established by Clustered Regularly Interspaced Short Palindromic Repeats/Cas9 synergistic activation mediator (CRISPR/Cas9–SAM) in HLE cells (Figure S1A–C, Supporting Information). Immunogold labeling revealed the presence of vWF on the surface of sEVs (Figure S1D, Supporting Information). To confirm the localization of vWF, proteinase K was used to degrade outer membrane‐localized proteins such as CD9 but not the intravesicular Alix and TSG101. The results showed that vWF was expressed on the surface of sEVs (Figure S1E, Supporting Information). Compared to control‐sEVs, vWF‐SAM‐sEVs augmented the formation of tubular structures and sprouts in HUVECs and microvessels in tumors (Figure 3F–H). Induced pulmonary vascular leakiness was detected in mice injected with vWF‐SAM‐sEVs, and the enhanced attachment of vWF‐SAM‐sEV‐treated HCC cells to HUVECs was observed (Figure 3I,J).

### 2.4 sEV–vWF Promotes HCC Tumorigenesis and Metastasis

**Figure 4:**
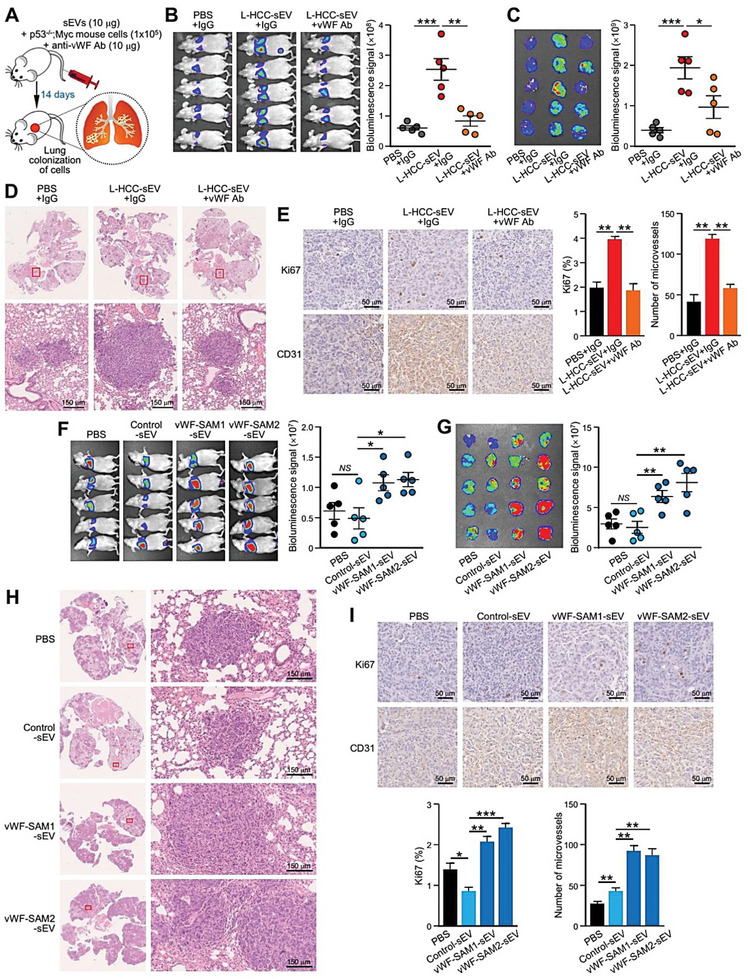
sEV–vWF promotes HCC metastasis. A) Schematic diagram of the experimental metastasis model. Luciferase‐labeled murine p53‐/‐;Myc hepatoblasts were co‐injected intravenously with or without L‐HCC‐sEVs together with either control IgG or anti‐vWF antibody into nude mice (*n* = 5 or 15 mice in total). B) Bioluminescence imaging of mice was performed 14 days post–injection. The intensity of the signal was quantified. C) Ex vivo bioluminescence imaging of the dissected lungs. The intensity of the signal was quantified. D) Representative images of H&E staining of the lung tissues after fixation. Enlarged images of the metastatic lesions depicted in the insets are shown. Scale bar, 150 µm. E) Immunohistochemistry of CD31 and Ki67 staining in lung metastases. F) Bioluminescence imaging of mice 14 days after intravenous co‐injection of luciferase‐labeled murine p53‐/‐;Myc hepatoblasts with either control‐sEVs or vWF‐SAM‐sEVs (*n* = 5 or 20 mice in total). G) Ex vivo bioluminescence imaging of dissected lungs. H) Representative images of metastatic lesions in lungs. Scale bar, 150 µm. I) Immunohistochemistry of CD31 and Ki67 staining in lung metastases. Data are presented as the mean ± SEM. *P < 0.05, **P < 0.01; and ***P < 0.001. *P* < 0.05 was regarded as statistically significant. NS, not significant.

In an experimental metastasis assay, mice injected with L‐HCC‐sEVs displayed increased colonization of murine p53‐/‐;Myc hepatoblasts in the lung compared to mice without sEV injection. This promoting effect was inhibited in mice injected with anti‐vWF antibody (**Figure** **4**A–D). Immunohistochemical staining revealed a significant increase in CD31 and Ki67 in lung metastases in mice injected with L‐HCC‐sEVs. The staining was reduced in mice treated with anti‐vWF antibody (Figure 4E). Consistently, vWF‐SAM‐sEVs potently augmented lung metastasis of murine p53‐/‐;Myc hepatoblasts (Figure 4F–H), and increased CD31 and Ki67 (Figure 4I). PLC/PRF/5 cells formed larger tumors when co‐injected with vWF‐SAM‐sEVs compared to cells co‐injected with control‐sEVs (Figure S2, Supporting Information). Taken together, these findings suggest that the augmented metastasis could be ascribed to sEV‐induced neovascularization and leakiness in the pulmonary vasculature to facilitate the dissemination of tumor cells through the hematogenous route and to assist the extravasation of tumor cells from the blood circulation to favorable distant sites.

### 2.5 sEV–vWF Modulates Endothelial Cells via Elevated Levels of VEGF‐A and FGF2

**Figure 5:**
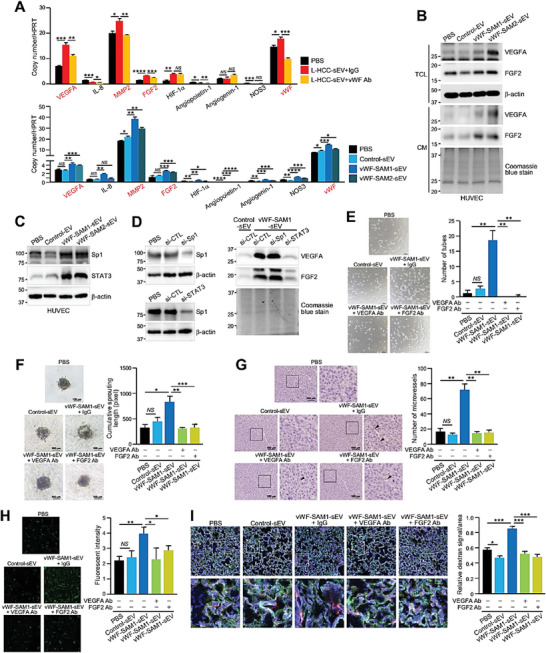
sEV–vWF modulates endothelial cells via elevated levels of VEGF‐A and FGF2. A) Quantitative PCR of proangiogenic genes in HUVECs treated with L‐HCC‐sEVs together with anti‐IgG or anti‐vWF antibody (upper panel) and HUVECs treated with control‐ and vWF‐SAM‐sEVs (lower panel) (*n* = 3). B) Western blotting of VEGF‐A and FGF2 expressions in the total cell lysate (TCL) and conditioned medium (CM) of HUVECs treated with control‐ and vWF‐SAM‐sEVs. C) Expressions of Sp1 and STAT3 in HUVECs treated with control‐ and vWF‐SAM‐sEVs were analyzed by immunoblotting. D) HUVECs transiently transfected with siRNA against Sp1 and STAT3 and subjected to western blot analysis (left panel). HUVECs transiently transfected with si‐Sp1 and si‐STAT3 and treated with vWF‐SAM‐sEVs were analyzed by western blot analysis (right panel). E) Tube formation (*n* = 3), F) endothelial sprouting (*n* = 5), and G) in vivo Matrigel plug angiogenesis (*n* = 3) assays were performed using HUVECs treated with control‐ and vWF‐SAM‐sEVs in the presence of either control IgG, anti‐VEGF‐A, or anti‐FGF2 antibody. H) Tumor–endothelial adhesion assays using PLC/PRF/5 cells and HUVECs treated with control and vWF‐enriched sEVs together with either control IgG, anti‐VEGF‐A, or anti‐FGF2 antibody (*n* = 3). I) Fluorescent image showing the vascular permeability of mouse lungs after intravenous co‐injection of Texas Red‐Dextran, FITC‐Lectin, and vWF‐enriched sEVs with antibodies against VEGF‐A or FGF2 (*n* = 3 or 15 mice in total). The Texas Red signal was quantified. Data are presented as the mean ± SEM. *P < 0.05, **P < 0.01; ***P < 0.001; and ****P < 0.0001. *P* < 0.05 was regarded as statistically significant. NS, not significant.

To explore how sEV–vWF modulates endothelial cells, we first examined whether HUVECs were able to internalize sEVs. The result showed that HUVECs internalized both control‐sEVs and vWF‐SAM‐sEVs with similar efficiency (Figure S3, Supporting Information). HUVECs treated with L‐HCC‐sEVs in the absence and presence of anti‐vWF antibody were analyzed for the expression of various proangiogenic factors. Four angiogenic factors, VEGF‐A, metalloproteinase 2 (MMP2), FGF2, and vWF, were elevated in HUVECs treated with L‐HCC‐sEVs, and their induction was suppressed by anti‐vWF antibody. This was corroborated by the induction of these angiogenic factors in HUVECs by vWF‐SAM‐sEVs but not by control‐sEVs (**Figure** **5**A). Among these four angiogenic factors, the sEV–vWF‐induced upregulation of VEGF‐A and FGF2 in the cell lysates and conditioned medium of HUVECs was confirmed by immunoblotting (Figure 5B). Sp1 and STAT3 have been implicated to be the transcriptional regulators of VEGF‐A and FGF2.^[^ ^18^ ^]^ It was found that both Sp1 and STAT3 expressions were induced by sEV–vWF in HUVECs (Figure 5C). To investigate whether the induction of VEGF‐A and FGF2 by sEV–vWF is driven by Sp1 and/or STAT3, Sp1 and STAT3 were suppressed and analyzed for the expressions of VEGF‐A and FGF2. The results showed that the knockdown of STAT3 but not Sp1 abrogated the induction of VEGF‐A and FGF2 induced by sEV–vWF, indicating STAT3 is the upstream regulator of VEGF‐A and FGF2 (Figure 5D). Functionally, when HUVECs were treated with anti‐VEGF‐A and anti‐FGF2 antibodies, the tube forming ability, and sprouting capabilities of HUVECs and the formation of microvessels in tumors induced by vWF‐SAM‐sEVs were largely compromised (Figure 5E–G), suggesting a role of VEGF‐A and FGF2 in endothelial vascularization. Apart from angiogenesis, the induction of tumor–endothelial adhesion and pulmonary leakiness by vWF‐SAM‐sEVs was also suppressed by anti‐VEGF‐A and anti‐FGF2 antibodies (Figure 5H,I). These results suggest that sEV–vWF‐induced endothelial cell release of VEGF‐A and FGF2 is involved in angiogenesis.

### 2.6 Feedback Response on HCC Cells by FGF2 Secreted by sEV–vWF‐Activated Endothelial Cells

**Figure 6:**
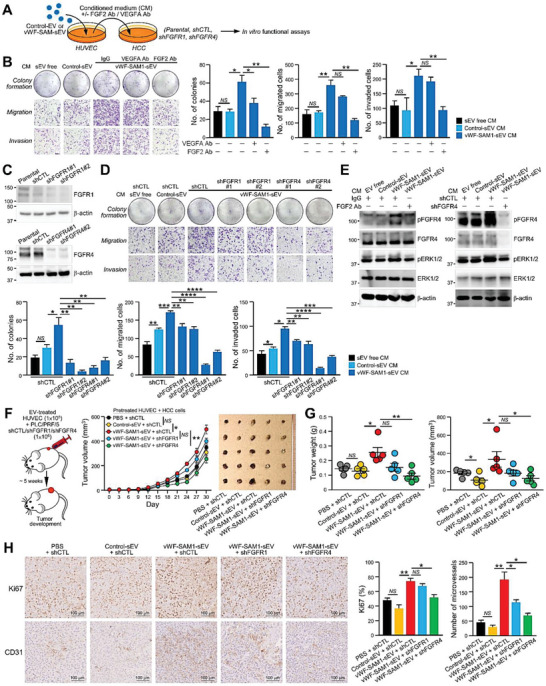
FGF2 secreted by sEV–vWF‐activated endothelial cells promotes colony formation and the migratory, invasive, and tumorigenic potential of HCC cells. A) Schematic diagram of the experimental setup of panels (B) and (D). B) Conditioned medium from HUVECs treated with the indicated sEVs was incubated with PLC/PRF/5 cells in the presence of VEGF‐A/FGF2 antibodies. Treated cells were subjected to colony formation, migration, and invasion assays (*n* = 3). The numbers of colonies and migrated and invaded cells were counted. C) Western blotting of the nontarget control (shCTL) and FGFR1 and FGFR4 of stable FGFR knockdown clones (shFGFR1 and shFGFR4) established using PLC/PRF/5 cells. D) Colony formation, migration, and invasion assays of shFGFR1 and shFGFR4 cells after incubation with conditioned medium collected from HUVECs treated with control‐ and vWF‐SAM‐sEVs (*n* = 3). E) PLC/PRF/5 cells treated with the indicated sEVs in the presence of IgG or FGF2 antibodies (left), and shCTL and shFGFR4 cells treated with the indicated sEVs (right) were subjected to immunoblotting. F) Monitoring the tumor growth derived from shFGFR1 and shFGFR4 cells co‐injected with HUVECs pretreated with the indicated EVs (left) (*n* = 5 or 25 mice in total); tumor size was measured regularly for 30 days (middle); and tumors were excised at the end of the experiment (right). G) The weight and volume of the tumors were measured and plotted. H) Immunohistochemistry of CD31 and Ki67 staining of the excised tumors. Data are presented as the mean ± SEM. *P < 0.05, **P < 0.01; ***P < 0.001; and ****P < 0.0001. *P* < 0.05 was regarded as statistically significant. NS, not significant.

According to the experimental setup in **Figure** **6**A, the conditioned medium from the control‐ and vWF‐SAM‐sEV‐treated HUVECs was found to induce the colony formation, migration, and invasion of PLC/PRF/5 and Huh7 cells, while such induction of colony‐forming ability was markedly suppressed with the addition of anti‐VEGF‐A and anti‐FGF2 antibodies. It was noted that their migratory and invasive potentials were only compromised by the anti‐FGF2 antibody (Figure 6B; Figure S4, Supporting Information). FGF2 belongs to the fibroblast growth factor family and exerts its effects by binding to four FGFR tyrosine kinase receptors (FGFR1–4).^[^ ^19^ ^]^ We wondered which FGFR was responsible for the effect of FGF2 in HCC cells; thus, the expression of FGFR copy number in various HCC cell lines was examined (Figure S5, Supporting Information). Among the four receptors examined, FGFR1 and FGFR4, which were expressed in most of the cell lines, were further analyzed by immunoblotting and functionally characterized. Nontarget control (shCTL) and FGFR knockdown clones (shFGFR1 and shFGFR4) were established in PLC/PRF/5 cells (Figure 6C). Notably, the migration and invasiveness of shFGFR4 cells treated with the conditioned medium of vWF‐SAM‐sEV‐treated HUVECs were largely compromised compare to shCTL and shFGFR1 cells (Figure 6D). vWF‐enriched sEV‐treated HUVEC‐conditioned medium activated phosphorylated FGFR4 and ERK expressions, which were downregulated by the addition of anti‐FGF2 antibody or FGFR4 knockdown in the recipient PLC/PRF/5 cells (Figure 6E). It was also found that FGF2–induced cellular vWF level and release of sEV–vWF by HCC cells was FGFR4 dependent, implicating a mutual stimulation between HCC and endothelial cells (Figure S6, Supporting Information). In the subcutaneous injection model, co‐injection with vWF‐SAM‐sEV‐treated HUVECs accelerated the tumor development of control PLC/PRF/5 shCTL cells but showed no effect on FGFR knockdown cells (Figure 6F,G). As indicated by the immunohistochemical staining of Ki67 and CD31, tumors activated by vWF‐SAM‐sEV‐treated HUVECs had the fastest cell proliferation and the most microvessel formation among all groups (Figure 6H).

**Figure 7:**
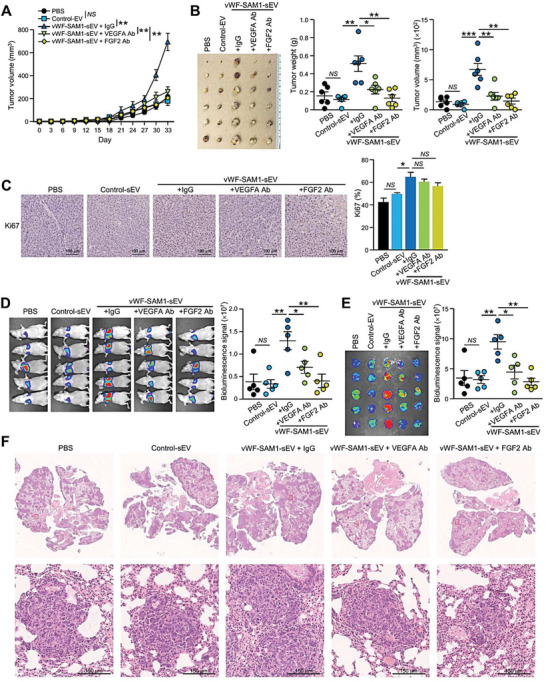
Anti‐VEGF‐A and FGF2 antibodies are effective in suppressing HCC tumorigenesis and metastasis. A) Tumor growth over time after subcutaneous co‐injection of PLC/PRF/5 cells with the indicated EVs and antibodies (*n* = 6 or 30 mice in total). B) Image of the excised tumors. Tumor weight and volume were measured. C) Immunohistochemical staining of Ki67 in the excised tumors. D) Luciferase‐labelled murine p53‐/‐;Myc hepatoblasts were co‐injected with the indicated sEVs and antibodies into the tail vein of mice (*n* = 5 or 25 mice in total). Bioluminescence imaging of mice was performed 14 days post–injection. The intensity of the signal was quantified. E) Ex vivo bioluminescence imaging of the dissected lungs. The intensity of the signal was quantified. F) Representative images of H&E staining of lung tissues after fixation. Enlarged images of the metastatic lesions are depicted in the insets. Scale bar, 150 µm. Data are presented as the mean ± SEM. *P < 0.05, **P < 0.01; and ***P < 0.001. *P* < 0.05 was regarded as statistically significant. NS, not significant.

The role of VEGF‐A and FGF2 in HCC growth and metastasis was further validated in vivo using neutralizing antibodies against VEGF‐A and FGF2. Significant retardation of vWF‐SAM‐sEV‐induced tumor growth and microvessel formation by anti‐VEGF and anti‐FGF2 antibodies compared to mice treated with control IgG was observed (**Figure** **7**A–C). Both neutralizing antibodies were also effective in suppressing lung colonization of murine p53‐/‐;Myc hepatoblasts induced by vWF‐SAM‐sEVs (Figure 7D–F).

**Figure 8:**
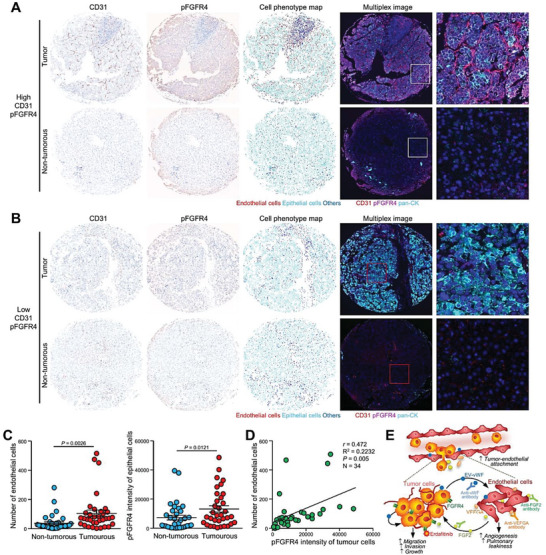
Clinical significance of angiogenesis and activation of FGFR4 in HCC. Multiplex IHC staining of CD31 and pFGFR4 was performed using tissue microarray that comprises 34 cases of paired HCC tissues and adjacent nontumorous tissues. Representative IHC staining of CD31 and pFGFR4, corresponding cell phenotype map, and multiplex merged images of A) HCC core with high level of CD31 and pFGFR4 (A) and B) of HCC core with low level of CD31 and pFGFR4. C) Fluorescent intensities of CD31 and pFGFR4 signals in tumorous and nontumorous tissues presented in a dot plot (*N* = 34). D) Correlation between the number of endothelial cells and pFGFR4 intensities in tumorous tissues analyzed by the Pearson correlation test. E) Schematic diagram of mechanism induced by sEV–vWF released by HCC cells. sEV–vWF induces elevation and secretion of VEGF‐A and FGF2 by endothelial cells, leading to promoted angiogenesis and vascular permeability. FGF2, in turn, activates FGFR4‐ERK1/2 axis to promote proliferation and motility of HCC cells.

Multiplex fluorescence immunohistochemical staining was performed to demonstrate the physiological relevance of feedback response revealed by the degree of angiogenesis and activation of FGFR4 in 34 cases of paired HCC tissues and adjacent nontumorous liver tissues (Figure S7, Supporting Information). As indicated by CD31 signal of endothelial cells, a higher number of endothelial cells were observed in tumorous tissues compared to nontumorous tissues. Higher activity of FGFR4 in tumorous tissues was shown by the more intense pFGFR4 signal than in nontumorous tissues (**Figure** **8**A,B). The number of endothelial cells and activation of FGFR4 in tumorous tissues was significantly correlated (Figure 8D). In summary, these findings reveal the role of VEGF‐A and FGF2 in angiogenesis and the crucial involvement of FGFR4 in the positive feedback signaling in HCC cells elicited by FGF2 and released by sEV–vWF‐activated endothelial cells (Figure 8E).

### 2.7 Targeting sEV–vWF with a Neutralizing Antibody and Erdafitinib Represents a Therapeutic Option for HCC

**Figure 9:**
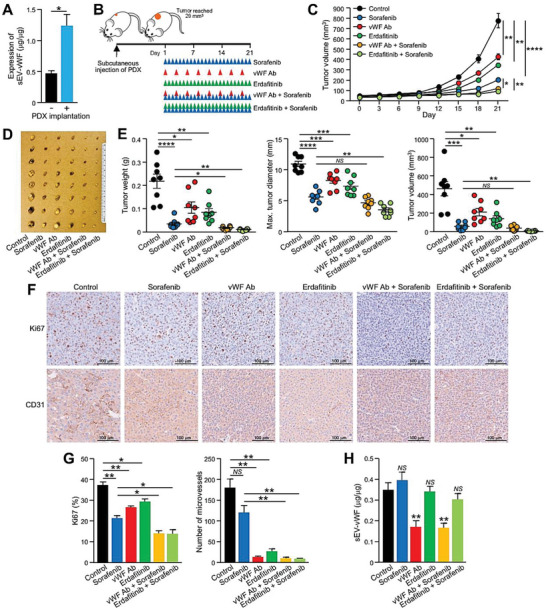
Anti‐vWF antibody and erdafitinib enhance the therapeutic efficacy of sorafenib in the growth of HCC patient‐derived tumor xenografts. A) ELISA of vWF in the circulating sEVs of mice with or without subcutaneous injection of HCC‐patient‐derived tumor xenografts (PDXs) (*n* = 3 or 6 mice in total). B) Schematic diagram of the therapeutic schedule in the HCC‐patient‐derived xenograft mouse model. C) Growth of subcutaneous PDXs over time in mice after treatment with sorafenib, an anti‐vWF antibody, erdafitinib, or both (*n* = 8 or 48 mice in total). D) Image of an excised tumor. E) Tumor weight, maximum dimension, and volume were measured. F) Immunohistochemistry of CD31 and Ki67 staining in the excised tumors. G) CD31 and Ki67 expression was quantified. H) ELISA of vWF in the circulating sEVs of mice after the treatment described in panel (B). Data are presented as the mean ± SEM. *P < 0.05, **P < 0.01; ***P < 0.001; and ****P < 0.0001. *P* < 0.05 was regarded as statistically significant. NS, not significant.

Our study demonstrated that the sEV–vWF/FGF2/FGFR4 cascade mediates tumor–endothelial cellular communication in HCC. The inhibitory effect of the anti‐vWF antibody in L‐HCC‐sEVs suggests that the neutralization of vWF‐enriched sEVs could be exploited as a therapeutic approach. Here, we questioned whether blockade of the sEV–vWF/FGF2/FGFR4 pathway alone or in combination with sorafenib, an Food and Drug Administration (FDA)‐approved drug for HCC patients, could be a better treatment for HCC patients. Subcutaneous injection using HCC‐patient‐derived xenografts (PDXs) was shown to express FGFR4 and resulted in an increase of circulating sEV–vWF in mice (**Figure** **9**A; Figure S8A, Supporting Information), revealing that this is a relevant mouse model to examine the therapeutic efficacy of anti‐vWF antibody, the pan‐FGFR inhibitor erdafitinib, and sorafenib (Figure 9B). Administration of the anti‐vWF antibody and erdafitinib significantly delayed tumor development and resulted in smaller tumors than those in untreated mice (Figure 9C–E). Co‐treatment of sorafenib with either anti‐vWF antibody or erdafitinib exerted the strongest suppressive effect. Immunohistochemistry identified significantly weaker CD31 and Ki67 staining of the excised tumors after co‐treatment (Figure 9F,G). A remarkable reduction in circulating sEV–vWF was detected in mice treated with anti‐vWF antibody (Figure 9H). All of these treatments were well tolerated in mice (Figure S8B, Supporting Information), suggesting their therapeutic potential for HCC patients (Figure 8C).

## 3 Discussion

vWF is a multimeric glycoprotein synthesized by endothelial cells, platelets, and sub‐endothelial connective tissues. It plays a pivotal role in platelet adhesion at the site of vascular injury and the formation of blood vessels.^[^ ^17^, ^20^ ^]^ Emerging evidence has revealed the involvement of vWF, apart from bleeding disorders, in various human cancers. High levels of plasma vWF have been reported in multiple hematological and nonhematological malignancies. Substantial elevation of vWF along with coagulation factor VIII has been found in lymphoma and leukemia patients.^[^ ^21^ ^]^ An abundance of vWF in blood is detected in gastric cancer^[^ ^14d^ ^]^ and is closely associated with cancer thromboembolism in malignant breast cancer.^[^ ^14c^ ^]^ Although high levels of vWF are frequently observed in a variety of cancers, a contradictory prognostic value of vWF has been noted. vWF reflects a poor prognosis of advanced metastatic colorectal cancer^[^ ^22^ ^]^ and gastric cancer,^[^ ^23^ ^]^ and is a biomarker of breast cancer progression.^[^ ^14c^ ^]^ The expression of vWF in tumors has also been suggested to be an indicator of activated endothelium or angiogenesis.^[^ ^13^ ^]^ Paradoxically, a high serum level of vWF has been reported to be a favorable prognostic factor in lung adenocarcinoma.^[^ ^24^ ^]^ These reports implicate the multifaceted roles of vWF in different cellular contexts. Intriguingly, emerging evidence has revealed the presence of vWF in sEVs and its involvement in diverse diseases. vWF is found in plasma‐derived sEVs from atrial fibrillation patients.^[^ ^25^ ^]^ In various types of cancers, including glioblastoma,^[^ ^26^ ^]^ oral squamous cell carcinoma,^[^ ^27^ ^]^ and chronic hepatitis‐related liver disease,^[^ ^16^, ^28^ ^]^ vWF has been shown to be upregulated in circulating sEVs. The rationale behind the dysregulation of vWF and its pathogenesis in HCC is far from clear. Here, we reveal the progressive upregulation of sEV–vWF during HCC development. The significant upregulation of sEV–vWF in patients with cirrhosis compared to control individuals indicates the potential of sEV–vWF as a noninvasive biomarker for the early detection of HCC.

Contrasting functional roles of exogenous vWF on angiogenesis have been discussed over the years. Starke et al. first reported antiangiogenic activity mediated by vWF, which leads to a reduction in in vitro tube formation ability.^[^ ^29^ ^]^ Enhanced vascularization is observed in vWF‐deficient mice, suggesting that plasma vWF is a key negative regulator of angiogenesis. In contrast, plasma vWF is capable of binding to multiple key growth factors to induce angiogenesis and catalyze wound healing.^[^ ^30^ ^]^ Under hindlimb ischemic conditions, ineffective vascularization was observed in vWF‐/‐mice.^[^ ^31^ ^]^ Tao et al. reported that vWF derived from tumor cells promotes angiogenesis in breast cancer.^[^ ^32^ ^]^ It is postulated that vWF^+^ sEVs determine the angiogenic activity after left ventricular assist device (LVAD) implantation.^[^ ^33^ ^]^

In the current study, we observed the proangiogenic ability of vWF, as the tube formation and sprouting abilities of HUVECs were significantly boosted after exposure to vWF‐enriched sEVs. Moreover, vWF possesses a strong adhesive ability that regulates homeostasis and platelet interactions. vWF could also link up platelets and tumor cells within the circulation to form heteroaggregates that develop resistance against anoikis and escape immune surveillance during the process of distal metastasis.^[^ ^34^ ^]^ It also facilitates cancer metastasis by accelerating the attachment of tumor cells to the microvasculature, which allows extravasation of tumor cells.^[^ ^35^ ^]^ In the same vein, we found that HCC‐derived sEV–vWF activates endothelial cells, aiding the attachment of tumor cells to the endothelium. We also demonstrated that sEV–vWF enhances angiogenesis, extravasation, and pulmonary colonization of cancer cells using a mouse model. In addition to the proangiogenic activity, sEV–vWF of HCC patient and cell lines was shown to increase HCC cell proliferation, migration, and invasiveness (Figures S9 and S10, Supporting Information). To the best of our knowledge, neither secreted vWF nor sEV‐derived vWF has been previously implicated in any mechanistic pathway in HCC. A study by Liu et al. reported that FGF2 enhances ONECUT2 expression via FGFR1/ERK/ELK1 signaling pathway in HCC cells. ONECUT2 induces transcription of FGF2 and ACLY expressions resulted in an enhanced HCC metastasis.^[^ ^36^ ^]^ Here, we revealed a crosstalk between HCC and endothelial cells. We showed the upregulation of the transcription, cellular level, and secretion of both VEGF‐A and FGF2 in recipient HUVECs activated by HCC‐derived sEV–vWF. Intriguingly, secreted FGF2 initiates feedback stimulation in HCC cells by binding to FGFR4, provoking HCC growth and metastasis through the activation of ERK signaling. At present, it remains obscure how vWF activates endothelial VEGF‐A and FGF2. It is essential to understand how sEV–vWF is taken up or binds to surface receptors of endothelial cells.

Last, this study demonstrated a significant blockade of circulating sEV–vWF by neutralizing antibodies could block HCC tumorigenicity and metastasis, demonstrating that neutralization of vWF can be exploited as a therapeutic approach. In a PDX mouse model, either an anti‐vWF neutralizing antibody or erdafitinib (a pan‐FGFR inhibitor) together with sorafenib improved treatment outcomes compared to sorafenib alone. Sorafenib, a multitarget tyrosine kinase inhibitor (TKI), is a first‐line treatment of HCC cells. Besides suppressing Raf‐mediated tumor proliferation, sorafenib exhibits anti–vascularization effects by inhibiting angiogenic stimulators including platelet‐derived growth factor receptors (PDGFR‐*β*) and vascular endothelial growth factor receptors (VEGFR).^[^ ^37^ ^]^ Lenvatinib, another first‐line TKI, has been shown to target aberrantly activated FGFR in HCC.^[^ ^38^ ^]^ These studies suggest targeting pathways involving VEGFR and FGFR could be beneficial for the treatment of HCC patients. According to our results, inhibiting sEV–vWF through neutralizing antibody significantly dampened the secretion of VEGF‐A and FGF2 in endothelial cells. The combination of anti‐vWF neutralizing antibody with sorafenib further limited PDX tumor growth, showing that the suppression of potent activators of VEGFR and FGFR could be a new strategy to improve the efficacy of sorafenib treatment in HCC. A report demonstrated that the blockade of FGF19/FGFR4 pathway significantly sensitizes sorafenib treatment toward HCC.^[^ ^39^ ^]^ Another study showed that DJ‐1/FGFR1 signaling pathway contributes to sorafenib resistance of HCC whereas FGFR1 affects the survival of sorafenib‐resistant HCC cells through the regulation of molecules involved in tumor apoptosis.^[^ ^40^ ^]^ In our PDX model, the administration of pan‐FGFR inhibitor Erdafitinib significantly improved the treatment outcome of sorafenib. Taken together, FGFR is implicated to be a strong regulator in the maintenance of HCC resistance against sorafenib, and targeting FGFR could potentially overcome such resistance.

## 4 Conclusion

To conclude, this study demonstrated a previously unknown function of vWF in angiogenesis and metastasis mediated by sEVs. We revealed the induction of vascularization by sEV–vWF through the upregulation and release of the proangiogenic factors VEGF‐A and FGF2 by endothelial cells. sEV–vWF‐activated endothelial FGF2 promotes tumor aggressiveness via positive feedback signaling toward HCC cells through an activated FGFR4‐ERK signaling axis. From a clinical perspective, vWF‐enriched sEVs are a potential biomarker for early diagnosis and a promising therapeutic target to achieve better clinical outcomes for HCC patients.

## 5 Experimental Section

### Human Samples

Serum samples were collected from healthy donors with a nonliver disease background (as control subjects), individuals with chronic hepatitis B virus (HBV) infection, and individuals with liver diseases (cirrhosis, early and late HCC) who had not received any treatment. Information about the serum donors is listed in Table S2 (Supporting Information). The collection of serum samples was conducted at Queen Mary Hospital, Hong Kong and Zhujiang Hospital, China, with informed consent from all donors. Fifty pairs of HCC tumorous and adjacent nontumorous liver tissues were surgically resected from patients at Queen Mary Hospital, Hong Kong. RNA was isolated from the tissues for the analysis of vWF expression. A tissue microarray consisting of paired tumorous and adjacent nontumorous liver tissues was constructed using tissue blocks from the Department of Pathology, Sun Yat‐sen University Cancer Centre, China. Procedure approval was sought from the Institutional Review Board of The University of Hong Kong/Hospital Authority Hong Kong West Cluster (HKU/HA HKW IRB), Zhujiang Hospital of Southern Medical University, and Sun Yat‐sen University Cancer Centre. The IRB reference numbers included UW 11–448, UW 17–211, and 2017‐GDEK‐003. All experiments involving human samples were handled in accordance with relevant ethical regulations.

### Animal Experimentation

Experimental procedures were performed under the research protocols CULATR 5530‐20 and 5950‐21 approved by the Committee of the Use of Live Animals in Teaching and Research (CULATR). All animal studies were conducted strictly according to the Animals (Control of Experiments) Ordinance (Hong Kong) and the Institute's guidance from the Centre for Comparative Medical Research (CCMR), Li Ka Shing Faculty of Medicine, The University of Hong Kong. All mice were provided by and housed in a specific pathogen‐free area in the CCMR building.

### Cell Culture

Human HCC cell line, PLC/PRF/5, and human embryonal kidney cells 293FT, were purchased from American Type Culture Collection (ATCC) and cultured according to the ATCC recommendations. For other HCC cell lines, Huh7 and HLE were obtained from Japanese Collection of Research Bioresources (JRCB, Japan), and MHCC97L and MHCCLM3 were obtained from Cancer Institute, Fudan University, China. Human immortalized normal liver cell lines MIHA was provided by Jayanta Roy‐Chowdhury, Albert Einstein College of Medicine, New York,^[^ ^41^ ^]^ and murine p53‐/‐; Myc hepatoblasts was provided by Scott Lowe, Memorial Sloan Kettering Cancer Center, New York.^[^ ^42^ ^]^ These cell lines were cultured according to provider's recommendations. Cell line was carried out by Short Tandem Repeat (STR) profiling. All cell lines were regularly tested to ensure absence of mycoplasma contamination.

### Isolation and Validation of sEVs

EV‐depleted fetal bovine serum (FBS) was obtained after overnight centrifugation at 100 000 × *g* at 4 °C (Beckman Coulter, Avanti JXN‐30). Cells were cultured in media supplemented with 10% EV‐depleted FBS for 72 h before sEVs were isolated by serial ultracentrifugation as described elsewhere.^[^ ^43^ ^]^ Briefly, culture supernatants were centrifuged at 3000 × *g* for 15 min to remove cell debris. Larger vesicles were removed after centrifugation at 20 000 × *g* for 30 min at 4 °C. Supernatants were then centrifuged at 100 000 × *g* for 75 min at 4 °C to pellet the sEVs. The sEVs were washed with PBS and collected by ultracentrifugation at 100 000 × *g* for 75 min at 4 °C. For circulating sEVs in human serum, 1 mL of serum was topped up to 30 mL by phosphate buffered saline (PBS) before subjecting to serial ultracentrifugation as described above. Mouse blood was obtained by cardiac puncture at the endpoint. Purification of circulating sEVs from mouse serum was performed using the ExoQuick PLUS Exosome Purification Kit for Serum & Plasma (System Biosciences). The serum was first centrifuged at 16 500 × *g* for 45 min (Eppendorf, 5430R) to pellet large vesicles. sEVs were then purified using the purification kit according to manufacturer's protocol. Lysed sEV proteins were subjected to immunoblotting using anti‐Alix (#2171, Cell Signaling Technology), anti‐CD9 (#ab92726, Abcam), anti‐TSG101 (#612 696, BD Biosciences), anti‐GM130 (#ab52149, Abcam), and anti‐p62 (#ab140651, Abcam) antibodies. ZetaView TWIN‐NTA PMX‐220 (Particles Metrix GmbH) was used to measure the particle size and concentration of sEVs according to the manufacturer's protocol. To examine the localization of vWF on or inside sEVs, 10 µg of sEVs was incubated with 1 µg of proteinase K (Thermo Fisher) for 30 min at 37 °C. The reaction was stopped by heating sEVs at 95 °C and mixed with 1× sodium dodecyl sulfate (SDS) for immunoblotting.

### Immunogold Labeling of sEVs

About 10 µL of collected sEVs was placed on formvar carbon‐coated nickel grids on parafilm for 20 min incubation. The grids were washed with PBS three times for 3 min. The grids were blocked with 1% bovine serum albumin (BSA) in PBS for 10 min. The grids were incubated with primary antibodies (1:25 ratio in 1% BSA) for 30 min, including anti‐CD63 (#ab134045, Abcam) and anti‐vWF (#sc‐53466, Santa Cruz Biotechnology) antibodies. The grids were washed with 1% BSA for 3 min three times and incubated for 20 min with 10 µL of gold‐conjugated secondary antibodies (5 or 15 nm) (1:1 ratio in 1% BSA) (#ab41498 and ab105290, Abcam). The grids were washed with PBS eight times before being counterstained with Reynold's lead citrate. The grids were visualized under Philips CM100 (Philips).

### Establishment of vWF Stable Clones

vWF was overexpressed in HLE cells using CRISPR SAM involving three vectors, Lenti dCAS‐VP64_Blast (#61 425, Addgene), LentiMPH v2 (#89 308, Addgene), and sgRNA(MS2)_zeo backbone (#61 427, Addgene) that carries sgRNA of vWF. sgRNA of vWF was subcloned into sgRNA(MS2)_zeo backbone via BsmBI site. The sequences of oligos of sgRNA are listed in Table S3 (Supporting Information). The successful subcloning of vWF sgRNA sequences into sgRNA(MS2)_zeo backbone was confirmed by DNA sequencing. Lentiviral plasmids were transfected into HEK293FT cells using Lenti‐Pac HIV Expression Packaging Kit (#LT001, GeneCopoeia) according to manufacturer's protocol. The viral supernatant was collected, centrifuged, and filtered before using for transduction of HCC cells. Polybrene (8 µg mL^−1^) (Sigma–Aldrich) was added as a transduction enhancer. HCC cells were selected by blasticidin (#A1113903, Thermo Fisher Scientific), puromycin (#A1113803, Thermo Fisher Scientific), and zeocin (#ant‐zn‐1, InvivoGen) according to the vector information. vWF‐SAM1, vWF‐SAM2 and respective Control clones were established.

### Establishment of FGFR Knockdown Stable Clones

FGFR1 and FGFR4 were knocked down in PLC/PRF/5 cells using short‐hairpin RNAs (shRNAs) targeting FGFR selected from RNAi Consortium library database and synthesized by Integrated DNA Technologies. The sequences of oligos are listed in Table S3 (Supporting Information). The duplexes were subcloned into pLKO.1‐Puro vector (#8453, Addgene) via AgeI and EcoRI sites. The presence of shRNA sequences in pLKO.1‐Puro vector was confirmed by DNA sequencing. The transduction procedure was the same as described above. FGFR1‐KD1, FGFR1‐KD2, FGFR4‐KD1, FGFR4‐KD2, and respective control clones were established.

### Treatment of Cells before Functional Assays

Cells were either pretreated with circulating sEVs or cell‐line‐derived sEVs before performing functional assays. Cells were seeded at a density of 3 × 10^4^ in 6‐well culture plates and treated with 2.5 µg mL^−1^ of sEVs for 72 h. To neutralize vWF, 1 µg mL^−1^ anti‐vWF neutralizing antibody (#SAB1408640, Sigma–Aldrich) was added to cell culture. To neutralize VEGF‐A and FGF2, 2 µg mL^−1^ anti‐VEGF‐A (#MAB293, R&D Systems) and anti‐FGF2 (#AF‐233, R&D systems) antibodies were added to cell culture. For control set‐up of neutralizing experiment, cells were treated with InVivoMAb polyclonal mouse IgG (#BE0093, BioXCell) or polyclonal rabbit IgG (BE0095, #BioXCell) using the same concentration as the tested experimental group.

### Colony Formation Assay

HCC cells were seeded at a density of 1 × 10^3^ cells per well in a 6‐well plate and incubated in a 37 °C incubator until colonies were visualized. Cells were fixed with methanol for 15 min and stained with crystal violet for 30 min. The number of colonies was counted.

### Migration and Invasion Assay

For migration assay, cells in serum‐free medium were seeded in the upper chamber of transwells. For invasion assay, BD Matrigel Basement Membrane Matrix (BD Bioscience) was used to coat Transwell Permeable Supports (Corning) before cell seeding. 3 × 10^4^ cells in serum‐free medium were seeded in the upper chamber of transwells. Dulbecco's Modified Eagle Medium (DMEM) supplemented with 10% FBS was added in the bottom chamber as a chemoattractant for both assays. After 16–18 h, cells were fixed with methanol for 1 h and stained with crystal violet for another 1 h. Four fields were randomly selected from each well, and the number of migrated and invaded cells was counted.

### Pulmonary Leakiness Assay

Six week old male BALB/cAnN‐nu mice were injected intravenously with 15 µg of sEVs or PBS as control. Twenty hours after sEV injection, mice were injected intravenously with Texas Red lysine‐fixable dextran (70 000 *M*_W_, Thermo Fisher Scientific) at 100 mg kg^−1^. After 3 h, mice were injected intravenously with 10 mg kg^−1^ Alexa Fluor concanavalin A (Thermo Fisher Scientific). Ten minutes later, each mouse was anesthetized and perfused with PBS and followed by 4% paraformaldehyde in PBS. Lung tissues were excised and immersed in 30% sucrose in PBS overnight. Tissues were cryosectioned with 12 µm thickness. Tissue sections were stained with 4′,6‐diamidino‐2‐phenylindole (DAPI, Thermo Fisher Scientific) and examined under confocal microscopy for vascular leakage indicated by Texas Red signal. Three random fields of each section were captured, and three sections per lung were examined. Images were processed and analyzed by ZEN software.

### Tube Formation Assay

About 100 µL of growth factor‐reduced Matrigel (Corning) was added to 24‐well plate on ice and allowed to solidify at 37 °C for 30 min. A total of 1 × 10^5^ HUVEC cells were seeded onto the coated surface and incubated for another 6 h at 37 °C. The angiogenic activity was assessed by counting the number of capillary tubes formed in three random fields under 4× objective lens.

### Sprouting Assay

About 1 mL of methocel stock solution (6 g methyl cellulose (Sigma–Aldrich) in 250 mL basal medium) was added to 4 mL of 1 × 10^5^ HUVEC cell suspension. A total of 120 drops with 25 µL each of the mixture were added onto the cover of the culture dish with multichannel micropipette. Hanging drops were incubated upside down in a humidified cell culture incubator for 24 h to allow spheroid formation. Prepare 4 mL collagen stock (Gibco) with 0.5 mL 10× Medium 199 (Gibco) on ice. pH value of the medium was adjusted with ice‐cold 0.2 n NaOH until collagen mixture turned to salmon pink color. Spheroid‐containing hanging drops were collected by washing with 10 mL of 1× PBS and centrifuged at 200 × *g* for 5 min. Cells were resuspended with solution containing 20% FBS methocel solution and collagen mixture at 1:1 ratio. About 1 mL of the spheroid–collagen mixture was added to each well of a 24‐well plate. The plate was incubated at 37 °C for 30 min to allow polymerization. About 100 µL of endothelial culture medium was added to each well to stimulate sprouting. The samples were fixed and analyzed with ImageJ.

### Adhesion Assay

HUVECs were seeded at 5 × 10^4^ per gelatin coated well in a 96‐well plate and incubated for 24 h until an endothelial monolayer was formed. The HUVEC monolayer was treated with tumor necrosis factor alpha (TNF‐*α*) for 6 h. 1 × 10^6^ of CytoTracker‐labeled HCC cells were washed twice with the serum‐free medium followed by centrifugation at 1000 rpm for 2 min. The cells were resuspended at 1 × 10^6^ cells mL^−1^. After removal of endothelial culture media, 2 × 10^5^ of cancer cell suspension was added into each well. Media were discarded after 1 h incubation and washed with 1× wash buffer gently for three times before discarding the final wash. To each well, 150 µL of 1× lysis buffer was added and incubated for 5 min at room temperature with shaking. About 100 µL of the lysed mixture was added to a 96‐well plate suitable for fluorescent measurement with a plate reader at 480 nm/520 nm.

### Matrigel Plug Angiogenesis Assay

1 × 10^6^ PLC/PRF/5 cells with or without sEVs were co‐injected (growth factor‐reduced Matrigel 4:1 medium) into 6 week old BALB/cAnN‐nu mice subcutaneously. The subcutaneous tumors were excised after 1 month for immunohistochemical staining using anti‐CD31 antibody (#ab28364, Abcam). The number of microvessels was counted.

### sEV Uptake Experiment

sEVs were labeled with PKH‐67 Membrane Dye Labeling Kit (Sigma–Aldrich) according to manufacturer's protocol with minor modifications. To assess the number of sEVs taken up by HUVECs, 5 × 10^4^ cells were seeded in 6‐well plate with cover slips for 24 h. An amount of 2.5 µg of PKH67‐labeled sEVs in serum‐free medium was added as treatment. After 2 h of incubation, sEV‐treated HUVECs were washed by PBS for five times followed by fixation with 4% paraformaldehyde and staining with phalloidin and DAPI. The coverslips were mounted onto slides and examined under laser scanning confocal microscopy. Images were analyzed using ImageJ. The relative PKH67‐sEVs uptake was determined by mean intensity of fluorescent signal from at least three different fields that were randomly selected from each sample.

### Subcutaneous Injection Assay

1 × 10^6^ PLC/PRF/5 cells with stimulators were co‐injected (Matrigel 4:1 medium) into 6week–old male BALB/cAnN‐nu mice subcutaneously. The tumor size and mice weight were monitored and recorded every 3 days after injection. At the end of experiment, the subcutaneous tumors were excised for the measurement of tumor weight and size, and histological analysis. All mice were included for data analysis.

### Experimental Metastasis Assay

For the lung colonization model, 1 × 10^5^ murine Myc‐transduced p53 null (p53‐/‐;Myc) hepatoblasts together with 10 µg sEVs or PBS were injected intravenously into 6 week old male BALB/cAnN‐nu mice. The mice were monitored by weekly bioluminescence imaging captured by IVIS spectrum imaging system (Perkin Elmer). At the end of experiment, ex vivo bioluminescence imaging of lungs was performed, and dissected lungs were subjected to histological analysis. All mice were included for data analysis.

### Co–treatment in Patient‐Derived Tumor Xenograft Model

Patient‐derived tumor xenografts were subcutaneously injected into BALB/cAnN‐nu mice. The growth of implanted tumors was monitored daily until it reached 20 mm^3^. Mice were randomly assigned to six groups with dimethyl sulfoxide (DMSO) in PBS (100 µL day^−1^; via intragastric injection), sorafenib (30 mg kg^−1^ day^−1^; by intragastric injection), Erdafitinib (30 mg kg^−1^ day^−1^; by intragastric injection), rabbit IgG antibody (5 µg per 3 days; via intraperitoneal injection), or anti‐vWF antibody (5 µg per 3 days; via intraperitoneal injection). The drug administration lasted for 21 days with the tumor size and mice body weight being measured every 3 days. The tumors were excised at the experimental endpoint, and the excised tumor volume, weight and maximum dimension were recorded. Tumors were sent for histological analysis. All mice were included for data analysis.

### Quantitative PCR Analysis

Total RNA was extracted from cells using RNAiso Plus (Takara) according to the manufacturer's instructions. Reverse transcription was performed using SuperScript VILO cDNA Synthesis Kit (Invitrogen). Real‐time polymerase chain reaction (PCR) was conducted using SYBR Green PCR Master Mix (Applied Biosystems) and performed on LightCycler 480 System (Roche Life Science). Sequences of oligos used in quantitative PCR (qPCR) are listed in Table S3 (Supporting Information).

### Western Blot Analysis

Protein lysates were obtained by cell lysis with radioimmunoprecipitation assay (RIPA) lysis buffer (Thermo Scientific), supplemented with 10% cOmplete protease inhibitor cocktail and 10% PhosStop phosphatase inhibitor cocktail (Roche Applied Science). Protein amount was quantified by BSA assay (Bio‐Rad Corporation). A total of 30 µg of protein per lane was resolved by 10% SDS–polyacrylamide gel electrophoresis (PAGE) (Bio‐Rad Corporation), followed by transferring to polyvinylidene fluoride (PVDF) membranes (Amersham) using Trans‐Blot Turbo System (Bio‐Rad Corporation). Chemiluminescent signals were detected by ECL Western Blotting Detection Reagents (GE Healthcare). Anti‐vWF (#sc‐53466, SantaCruz Biotechnology), anti‐*β*‐actin (#A5316, Sigma–Aldrich), anti‐VEGF‐A (#ab46154, Abcam), anti‐FGF2 (#ab208687), anti‐FGFR1 (#ab76464, Abcam), anti‐FGFR4 (#ab178396, Abcam), anti‐phospho‐FGFR4 (#ab192589, Abcam), anti‐phospho‐ERK1/2 (#9101, Cell Signaling Technology), anti‐ERK1/2 (#4695, Cell Signaling Technology), anti‐Sp1 ((#ab13370, Abcam), and anti‐STAT3 ((#12 640, Cell Signaling Technology) antibodies were used for immunoblotting.

### Immunohistochemistry and Hematoxylin/Eosin (H&E) staining

Formalin‐fixed, paraffin‐embedded tissue was sectioned with a thickness of 5 µm and deparaffinized in xylene, followed by rehydration in a gradient of alcohols (100%, 95%, and 70%) and distilled water. Antigen retrieval was conducted by immersing the sections in preheated EnVision FLEX Target Retrieval Solution, High pH (Agilent). The sections were microwaved for a further 15 min and allowed to cool down in the retrieval solution for at least 20 min at room temperature. After blocking the endogenous peroxidase by EnVision FLEX Peroxidase‐Blocking Reagent, the sections were subjected to incubation with primary and secondary antibodies. Signal detection was facilitated by the addition of Dako REAL EnVision Detection System and 3,3'–Diaminobenzidine (DAB) chromogen for 30 and 2 min, respectively, at room temperature. The specimen section was also stained with hematoxylin and eosin stain. NanoZoomer Digital Pathology System (Hamamatsu) was used to process slides and to create high‐quality digital images for analysis. Anti‐CD31 (#ab28364, Abcam) and anti‐Ki67 (#M7240, Dako) antibodies were used for immunohistochemical staining. The signal was quantified by counting the number of positively stained cells against total number of cells in three different fields randomly selected in each sample.

### Multiplex Fluorescent Immunohistochemistry Staining

Tissues of tissue microarray (TMA) were sliced into sections with 9 µm thickness and mounted onto glass slices. Multiplex immunofluorescent staining was performed on TMAs using Opal 7‐Color IHC kits (Akoya Biosciences, Hopkinton, MA) according to the manufacturer's instructions. Briefly, sections were deparaffinized using xylene and progressively hydrated by ethanol ending with a distilled water wash. Microwave treatment was performed in AR6 buffer for antigen retrieval. Then slides were sequentially stained with the following primary antibodies and fluorescent dyes: anti‐CD31 (1:50, #ab9498, Abcam)/Opal‐520, Anti‐phospho‐FGFR4 (1:200, #PA5105531, Invitrogen)/Opal‐620, anti‐pan‐cytokeratin (1:100, #ab86734, Abcam)/Opal‐690. For each molecule being detected, slides were incubated with the specific primary  and secondary antibody, followed by Opal fluorophore solution and incubated for 10 min at room temperature. Afterward, microwave treatment was performed again to remove the specific primary antibody. Steps were repeated for subsequent primary antibodies to achieve multiplex IF staining. After antibody staining, slides were incubated in DAPI solution for 5 min at room temperature, washed several times, and then mounted with coverslips. Digital images of all cores of TMA were acquired using the Vectra Polaris Imaging System and analyzed by inForm software (Akoya Biosciences).

### Enzyme‐Linked Immunosorbent Assay

Human vWF enzyme‐linked immunosorbent assay (ELISA) kit (ab108918, Abcam) was used to determine vWF level in sEVs extracted from sera of mouse and patients as well as cell culture medium according to manufacturer's protocol. The isolated sEVs were lysed, and the proteins were subjected to the measurement of vWF. The level of sEV–vWF was expressed as the amount of vWF over sEV protein amount (µg µg^−1^) (w/w).

### Statistical Analysis

All data were calculated as the mean ± standard error of the mean (SEM). Statistical analysis was performed using a *t*‐test and Analysis of Variance (ANOVA) by Prism (Version 8.0.1, GraphPad). A *P‐*value of less than 0.05 was considered statistically significant.

## Conflict of Interest

The authors declare no conflict of interest.

## Author Contributions

Conceptualization: S.W.K.W. and J.W.P.Y. Methodology: S.W.K.W. and J.W.P.Y. Investigation: S.W.K.W., S.K.T., X.M., H.L.F., and Z.‐J.X.; Resources: D.K.H.W., L.‐Y.M., M.‐F.Y., I.O.‐L.N., J.P.Y., Y.G., and J.W.P.Y. Funding acquisition: J.W.P.Y. Supervision: J.W.P.Y. Writing—original draft: S.W.K.W. and J.W.P.Y. Writing–review and editing: S.W.K.W. and J.W.P.Y.

## Supporting information

Supporting Information

## Data Availability

The data that support the findings of this study are available from the corresponding author upon reasonable request.
