# Correction to “Small Extracellular Vesicle‐Derived vWF Induces a Positive Feedback Loop between Tumor and Endothelial Cells to Promote Angiogenesis and Metastasis in Hepatocellular Carcinoma”

**DOI:** 10.1002/advs.202416993

**Published:** 2025-01-13

Adv Sci (Weinh). 2023 Jun 30;10(26):2302677

https://doi.org/10.1002/advs.202302677

In Figure 5G, the image of Control‐sEV was erroneously duplicated for vWF‐SAM1‐sEV + FGF2 Ab. We kindly ask for Figure 5G to be corrected as follows:

Corrected Figure 5G


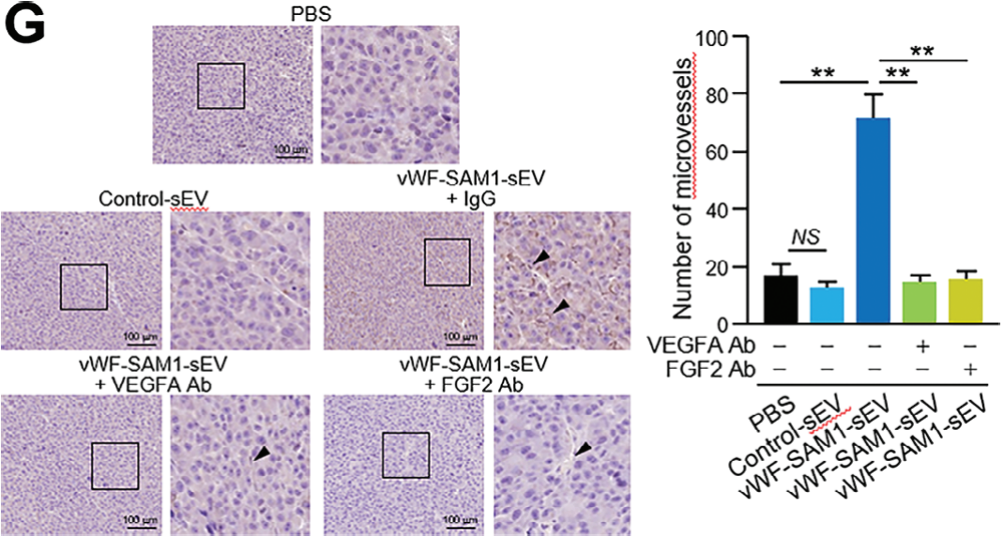


In Figure 5I, the immunofluorescence images of vWF‐SAM1‐sEV + VEGFA Ab and vWF‐SAM1‐sEV + FGF2 Ab were mistakenly represented as images of PBS. We kindly request for Figure 5I to be corrected as follows:

Corrected Figure 5I


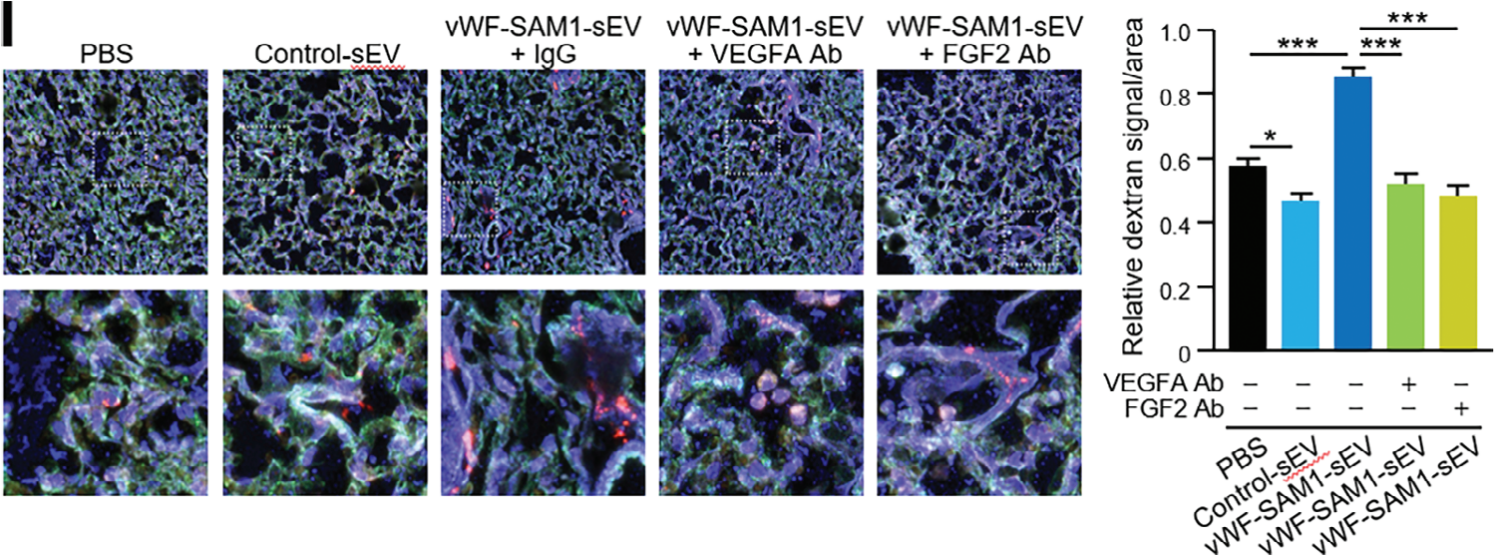


The same data was mistakenly presented in Figure 3H and Supplementary Figure 2E. Both the representative images and bar charts are identical, although different areas were captured for the enlarged image. We therefore request to omit Supplementary Figure 2E.

These corrections do not impact the overall findings and conclusions of the paper.

We apologize for these errors.

