# Supplementary material for: Small Extracellular Vesicle‐Derived vWF Induces a Positive Feedback Loop between Tumor and Endothelial Cells to Promote Angiogenesis and Metastasis in Hepatocellular Carcinoma
Source: Adv Sci (Weinh). 2023 Jun 30;10(26):2302677. doi: 10.1002/advs.202302677 (PMC10502836; doi:10.1002/advs.202302677)
Supplement: Supplementary file 1 — Supporting Information [file ADVS-10-2302677-s001.pdf]

ADVANCED SCIENCE
Open Access

Supporting Information

for *Adv. Sci.*, DOI 10.1002/advs.202302677

Small Extracellular Vesicle-Derived vWF Induces a Positive Feedback Loop between Tumor and Endothelial Cells to Promote Angiogenesis and Metastasis in Hepatocellular Carcinoma

*Samuel Wan Ki Wong, Sze Keong Tey, Xiaowen Mao, Hiu Ling Fung, Zhi-Jie Xiao, Danny Ka Ho Wong, Lung-Yi Mak, Man-Fung Yuen, Irene Oi-Lin Ng, Jing Ping Yun, Yi Gao and Judy Wai Ping Yam**

Supporting Information

Small extracellular vesicle-derived vWF induces a positive feedback loop between tumour and endothelial cells to promote angiogenesis and metastasis in hepatocellular carcinoma

*Samuel Wan Ki Wong, Sze Keong Tey, Mao Xiaowen, Hiu Ling Fung, Zhi-Jie Xiao, Danny Ka Ho Wong, Lung-Yi Mak, Man-Fung Yuen, Irene Oi-Lin Ng, Jing Ping Yun, Yi Gao, Judy Wai Ping Yam**

Supplementary Figures

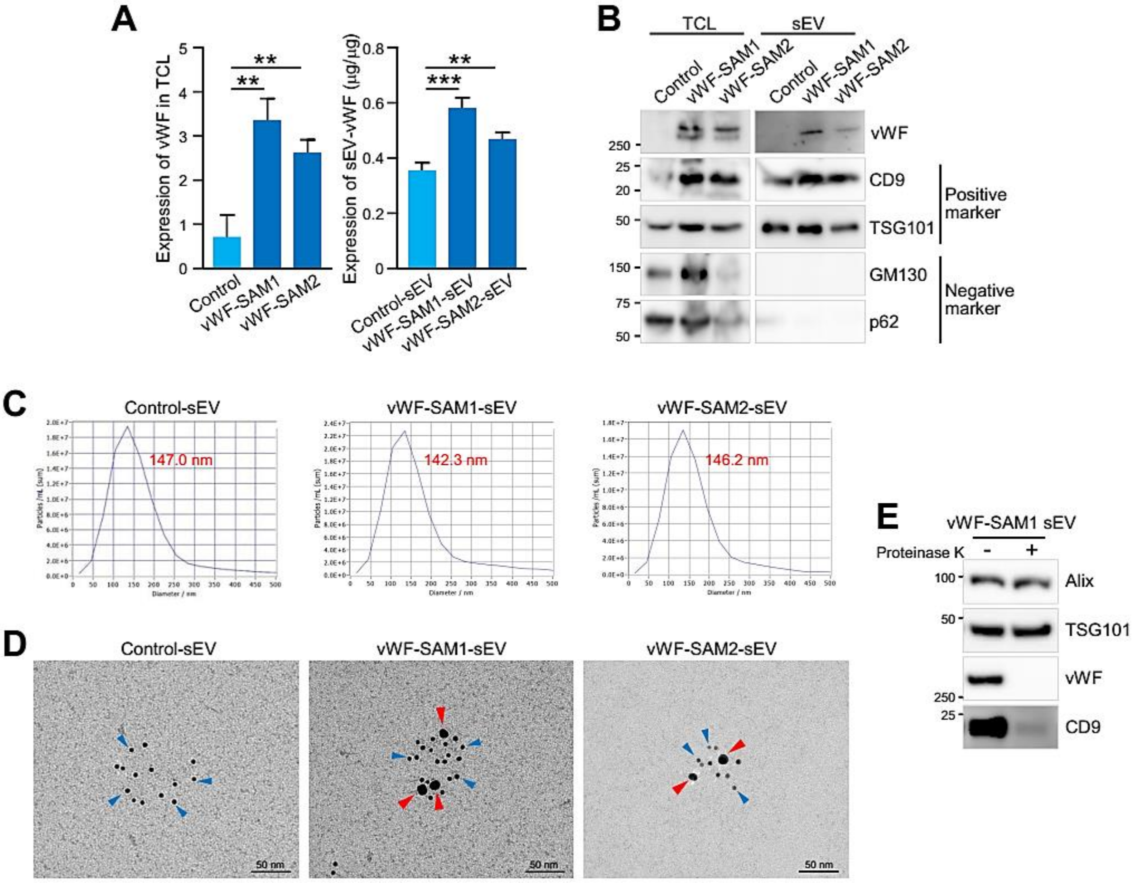

Supplementary Figure 1. Characterisation of vWF-enriched sEV. A. ELISA of vWF level in sEV collected from the conditioned medium of HLE control (Control-sEV) and vWF overexpressing (vWF-SAM1-sEV and vWF-SAM2-sEV) cells. **B.** Western blotting of vWF, positive and negative sEV markers in the total cell lysate (TCL) and isolated Control-sEV and vWF-SAM1/2-sEV of HLE cells. **C.** Size distribution of the indicated sEV measured by ZetaView® TWIN-NTA PMX-220. **D.** Representative electron micrographs of the indicated sEV co-stained by anti-CD63 and anti-vWF antibodies and secondary antibody conjugated with 5 nm and 15 nm gold particles, respectively. Red arrowhead indicates vWF and blue arrowhead indicates CD63. Scale bar, 50 nm. **E.** Western blot analysis showing expressions of Alix, TSG101, vWF and CD9 in vWF-SAM1-sEV treated with or without 1 μg/ml proteinase K. Data are presented as mean ± SEM. $^{**}P < 0.01$; $^{***}P < 0.001$. $P < 0.05$ is regarded as statistically significant. *NS*, not significant.

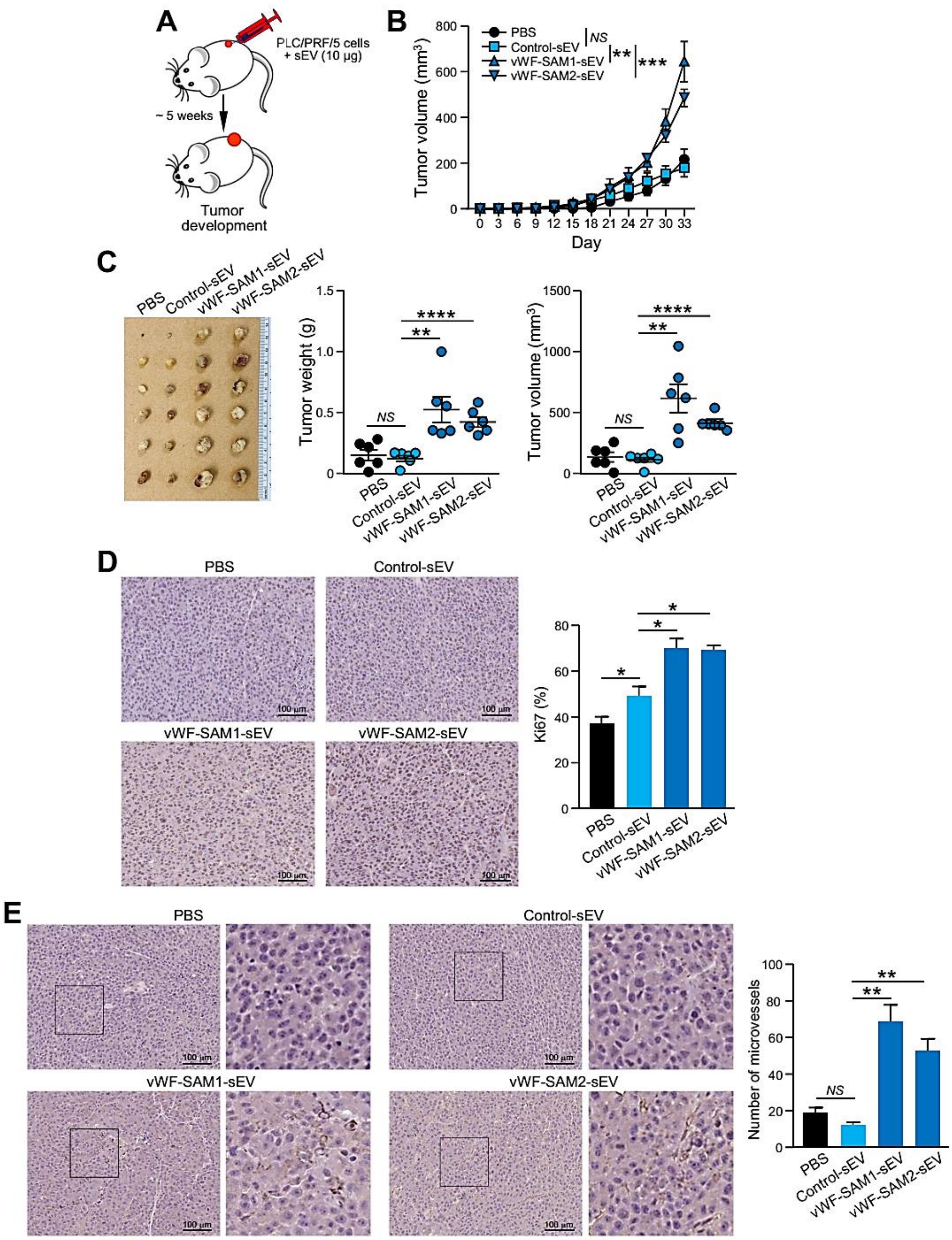

Supplementary Figure 2. sEV-vWF promotes HCC tumourigenesis. A. Schematic diagram of *in vivo* tumourigenicity model. PLC/PRF/5 cells were subcutaneously co-injected with sEVs into BALB/c-nu mice. The tumour growth was monitored for 5 weeks. Tumour growth over time **(B)**, excised tumour images, weight and volume **(C)** are shown. Immnuohistochemical staining of Ki67 **(D)** and CD31 **(E)** of excised tumour (n = 6). Signals of Ki67 and CD31 were quantified. Data are presented as mean ± SEM. $*P < 0.05$; $**P < 0.01$; $****P < 0.0001$. $P < 0.05$ is regarded as statistically significant. *NS*, not significant.

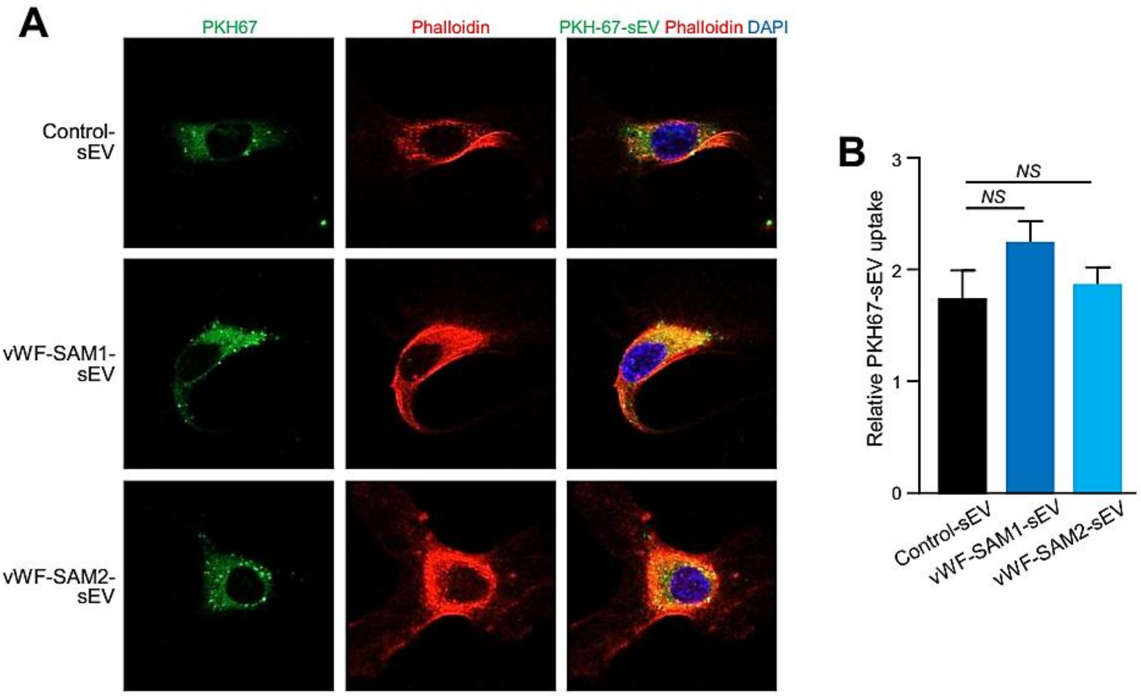

Supplementary Figure 3. Uptake of sEV by endothelial cells. A. HUVECs were incubated with PKH67-labeled sEVs collected from the conditioned medium of HLE control (Control-sEV) and vWF overexpressing (vWF-SAM1-sEV and vWF-SAM2-sEV) cells. After incubation, cells were stained by phalloidin and DAPI. Scale bar: 20 µm. Representative images are shown. B. Data are expressed as arbitrary units and are presented relative to the value obtained for cells treated with Control-sEV.

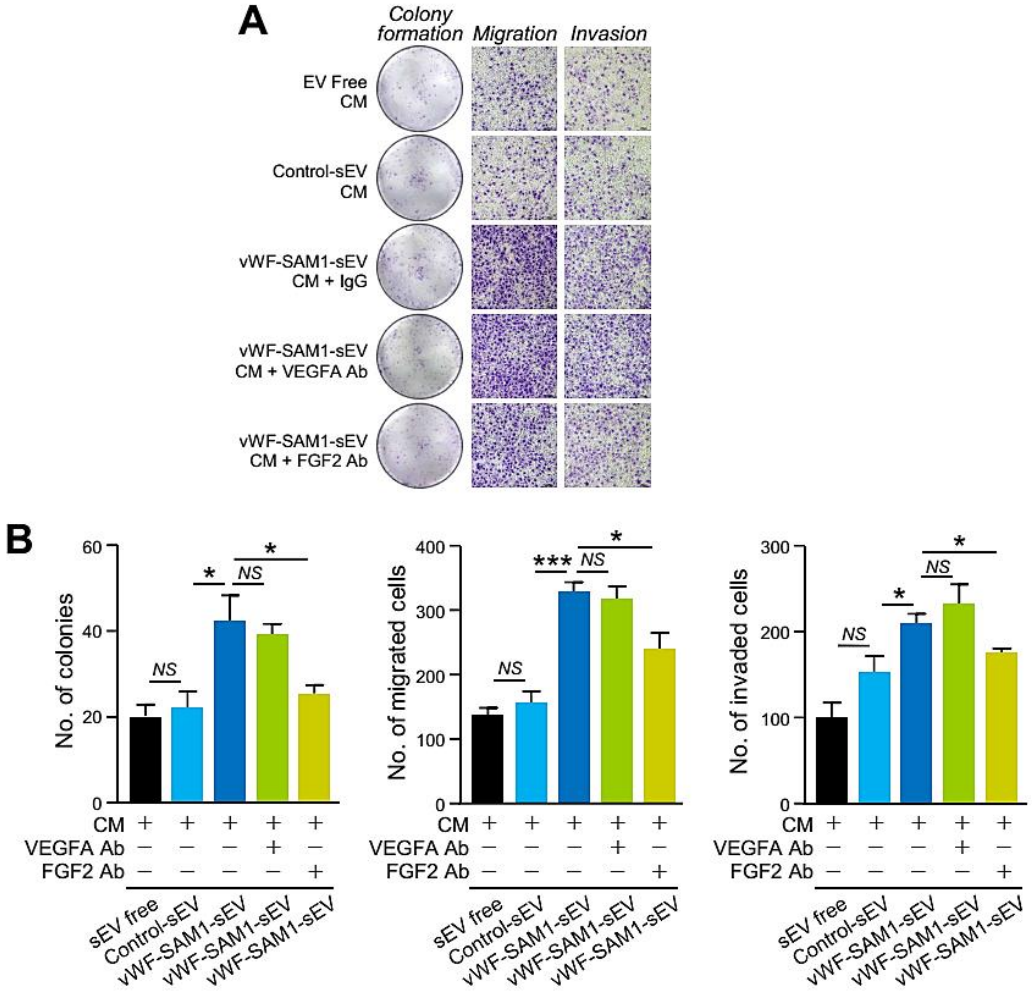

Supplementary Figure 4. Anti-VEFGA and anti-FGF2 antibodies inhibit sEV-vWF-induced cell growth and motility. A. Representative images of colony formation, migration and invasion assays of Huh7 after incubation with conditioned medium of HUVEC pre-treated with sEV in the presence of anti-VEGFA or anti-FGF2 antibodies. B. Calculated number of colonies, migrated cells and invaded cells. Data are presented as mean ± SEM. $*P < 0.05$; $***P < 0.001$. $P < 0.05$ is regarded as statistically significant. *NS*, not significant.

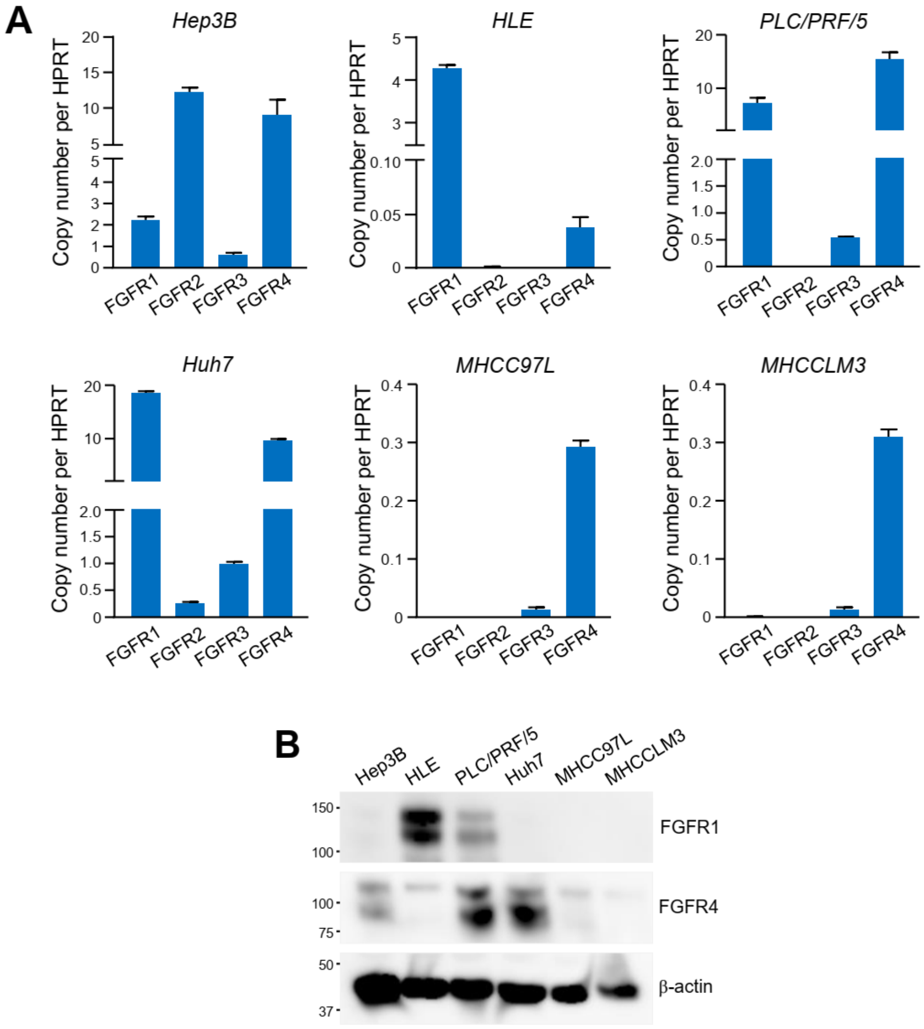

Supplementary Figure 5. Expression of FGFR in HCC cell lines. A. Quantitative PCR of FGF receptors in cell line, Hep3B, HLE, Huh7, PLC/PRF/5, MHCC97L and MHCCLM3. **B.** Expression of FGFR1 and FGFR4 in HCC cell lines was analyzed by immunoblotting.

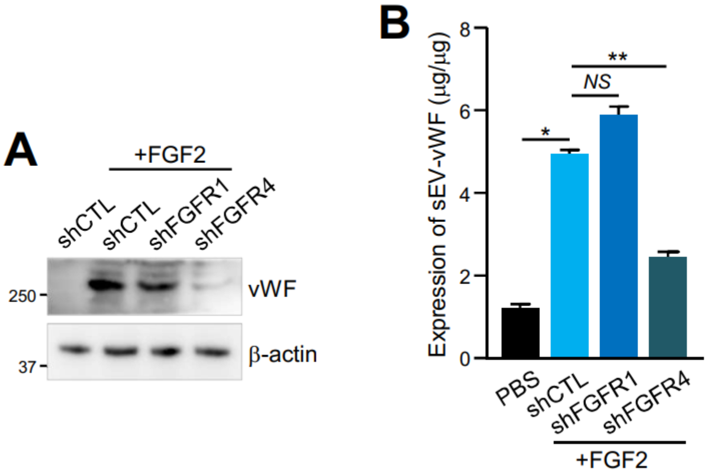

Supplementary Figure 6. FGF2 induces cellular level of vWF and release of sEV-vWF by HCC cells. A. PLC/PRF/5 non-target control, FGFR1 and FGFR4 knockdown cells treated with FGF2 recombinant protein were examined for vWF expression by western blot analysis. **B.** Conditioned medium of PLC/PRF/5 non-target control, FGFR1 and FGFR4 knockdown cells treated with FGF2 recombinant protein were collected for sEV isolation. The level of vWF of isolated sEV was analysed by ELISA. Data are presented as mean ± SEM. *$P < 0.05$; **$P < 0.01$. $P < 0.05$ is regarded as statistically significant. *NS*, not significant.

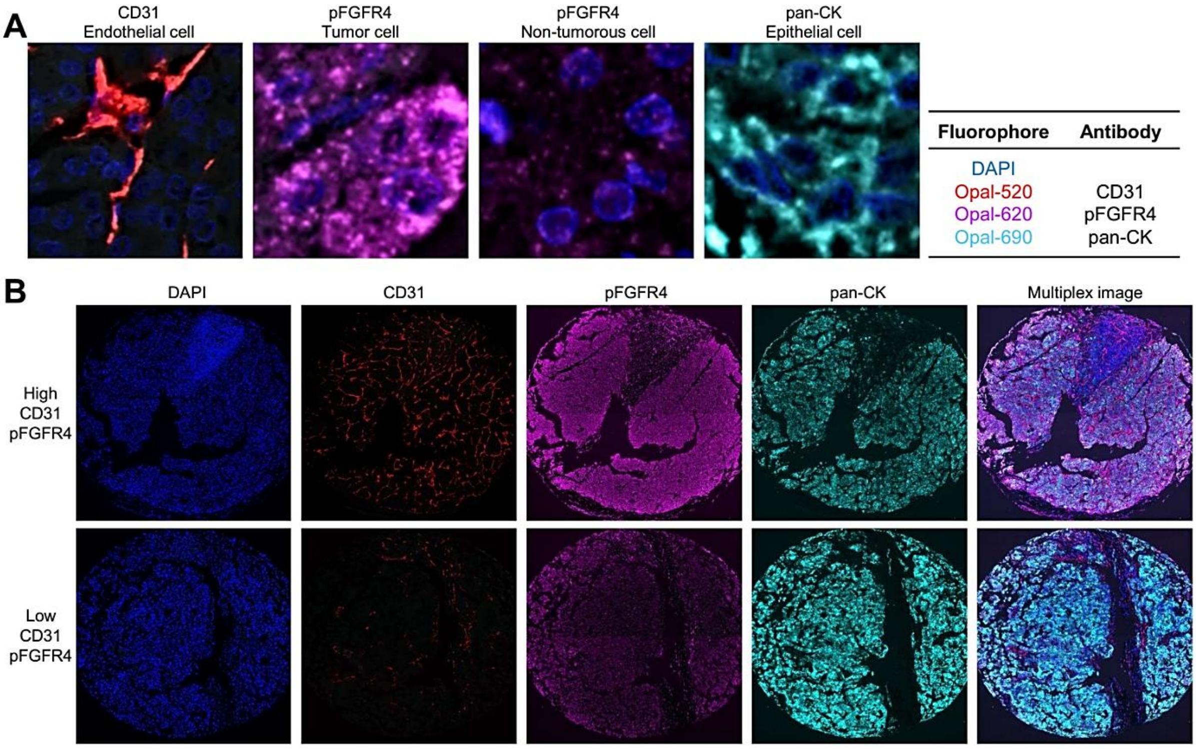

Supplementary Figure 7. Correlation between CD31 and pFGFR4 was analysed by 4-color multiplex fluorescent immunohistochemistry. A. Images were scanned and analysed by Vectra Polaris system and inform software, respectively. Representative images for cell phenotyping. Endothelial cell was defined by CD31, epithelial cell was defined by pan-cytokeratin (pan-CK), and tumour cell was defined by the size of nuclear DAPI signal. **B.** Representative single colour images and merge image of DAPI, CD31, pFGFR4 and pan-CK staining of HCC core with high intensity of both CD31 and pFGFR4 (*upper panel*) and of HCC core with low intensity of both CD31 and pFGFR4 (*lower panel*).

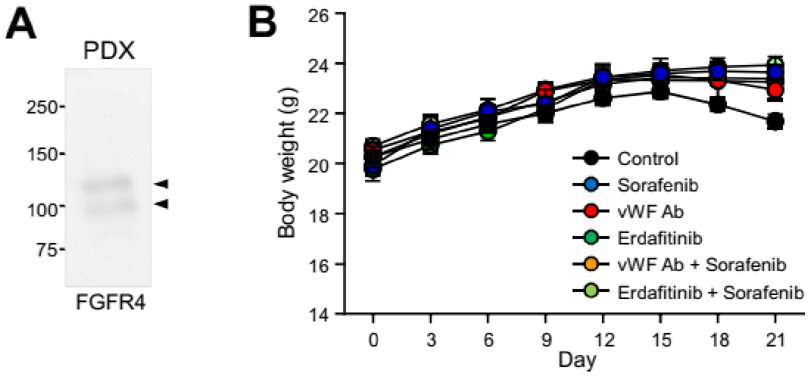

Supplementary Figure 8. Patient-derived xenograft mouse model. A. Western blotting of FGFR4 expression in HCC patient-derived xenograft (PDX)**. B.** Body weight of mice was examined every 3 days till the experimental endpoint. Data was plotted against time.

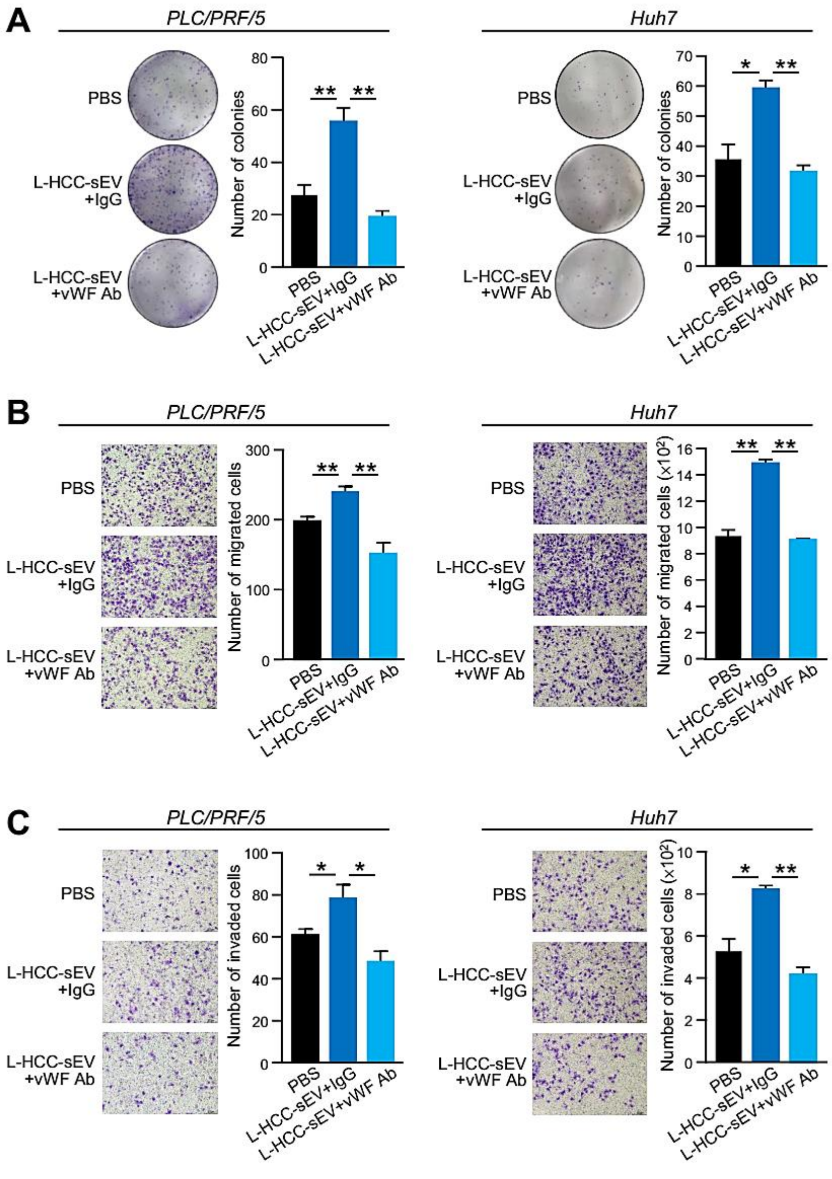

Supplementary Figure 9. Anti-vWF antibody inhibits promotion of cell growth and motility induced by HCC patient circulating sEV. PLC/PRF/5 (*left*) and Huh7 (*right*) cells were incubated with L-HCC-sEV in the presence of control IgG or anti-vWF antibody. The treated cells were subjected to colony formation **(A)**, migration **(B)** and invasion assays **(C)**. The number of colonies and cells were counted. Data are presented as mean ± SEM. $^{*}P < 0.05$; $^{**}P < 0.01$. $P < 0.05$ is regarded as statistically significant.

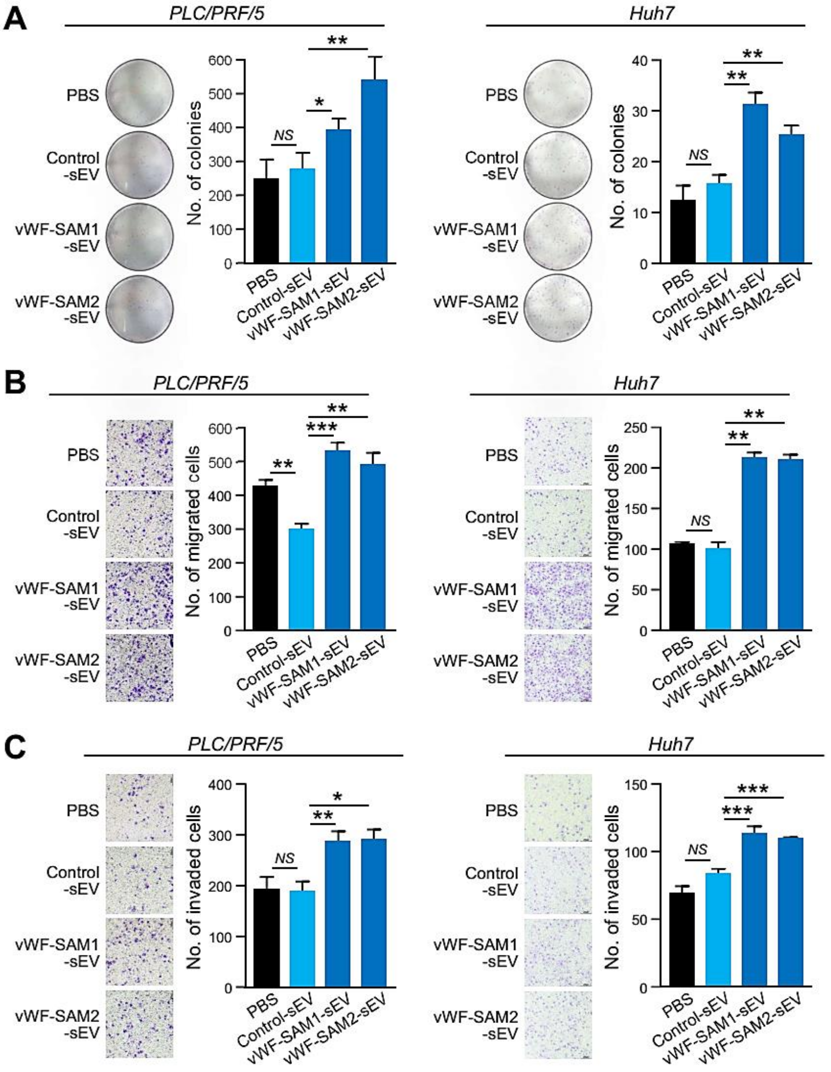

Supplementary Figure 10. vWF-enhanced sEV promotes HCC cell growth and motility. PLC/PRF/5 (*left*) and Huh7 (*right*) cells were incubated with either control- or vWF-SAM-sEV. The treated cells were subjected to colony formation **(A)**, migration **(B)** and invasion assays **(C)**. The number of colonies and cells were counted. Data are presented as mean ± SEM. **P* < 0.05; ***P* < 0.01, ****P* < 0.001. *P* < 0.05 is regarded as statistically significant. *NS*, not significant.

Supplementary Table 1. Expression of top 10 upregulated proteins of L-HCC-sEV compared to Normal-sEV obtained from mass spectrometry

	Mean reading				
Protein name	Normal -sEV	Cirrhosis -sEV	E-HCC -sEV	L-HCC -sEV	*Fold change
IGHG1	161615066	90637574	357403449	31708545878	196.20
pIgR	49684579	54326437	35880628	919979586	18.52
IGHG3	112067585	416131101	306112703	1166513546	10.41
LGALS3BP	160560131	260269348	866534284	1636105017	10.19
IGLC6	4282351576	2852252410	194968292	41784975114	9.76
CP	115631870	170259741	88250031	903849835	7.82
vWF	293994940	219845011	418807349	1607454220	5.47
IGHA1	39919473291	45559991712	1057699032	209285035471	5.24
IGHV5-51	79167396	52325940	21096189	325359719	4.11
SERPINA1	208504499	168769913	74570119	421509339	2.02

* Comparison between L-HCC-sEV and Normal-sEV

Supplementary Table 2. Information of serum donors

	Clinical parameters	Category	Number of donors
**HCC patients**	Gender	Male	45
		Female	10
	Age	< 50	17
		50 - 60	17
		> 60	21
	HBsAg	Positive	35
		Negative	20
	Cirrhotic liver	Cirrhosis	25
		Normal and CH	30
	pTNM stage	I and II	37
		III and IV	18
	**Clinical parameters**	**Category**	**Number of donors**
**Individuals with chronic HBV infection and cirrhosis**	Gender	Male	28
		Female	0
	Age	< 50	7
		50 - 60	2
		> 60	19
	**Clinical parameters**	**Category**	**Number of donors**
**Control individuals**	Gender	Male	9
		Female	9
	Age	< 50	4
		50 - 60	8
		> 60	6

HBsAg, Hepatitis B surface antigen; CH, Chronic hepatitis; pTNM = Pathological tumour-node-metastasis

Supplementary Table 3. Oligonucleotides used in the study

Name of oligos	Sequence 5' to 3'
VWF-qF	CCGATGCAGCCTTTTCGGA
VWF-qR	TCCCCAAGATACACGGAGAGG
vWF-SAM-1F	CACCGAAGTGTGGGGGTGTAGGGAT
vWF-SAM-1R	AAACATCCCTACACCCCCACACTTC
vWF-SAM-2F	CACCGAGTTAATTAAGGCAGCTGCC
vWF-SAM-2R	AAACGGCAGCTGCCTTAATTAACTC
VEGFA-qF	AGGGCAGAATCATCACGAAGT
VEGFA-qR	AGGGTCTCGATTGGATGGCA
IL-8-qF	TTTTGCCAAGGAGTGCTAAAGA
IL-8-qR	AACCCTCTGCACCCAGTTTTC
MMP2-qF	TACAGGATCATTGGCTACACACC
MMP2-qR	GGTCACATCGCTCCAGACT
FGF2-qF	AGAAGAGCGACCCTCACATCA
FGF2-qR	CGGTTAGCACACACTCCTTTG
HIF-1α-qF	GAACGTCGAAAAGAAAAGTCTCG
HIF-1α-qR	CCTTATCAAGATGCGAACTCACA
Angiopoietin-1-qF	AGTGGACACTGGACATTGCAG
Angiopoietin-1-qR	GCTTCCTCTTTACCATCTGTGG
Angiogenin-1-qF	CTGGGCGTTTTGTTGTTGGTC
Angiogenin-1-qR	GGTTTGGCATCATAGTGCTGG
NOS3-qF	TGATGGCGAAGCGAGTGAAG
NOS3-qR	ACTCATCCATACACAGGACCC
HPRT1-qF	CAGCCATGAGAAGTGGTTCTGT
HPRT1-qR	CACTGATGAAGCACAGCCCTTA
FGFR1-qF	CCCGTAGCTCCATATTGGACA
FGFR1-qR	TTTGCCATTTTTCAACCAGCG
FGFR2-qF	AGCACCATACTGGACCAACAC
FGFR2-qR	GGCAGCGAAACTTGACAGTG
FGFR3-qF	TGCGTCGTGGAGAACAAGTTT
FGFR3-qR	GCACGGTAACGTAGGGTGTG
FGFR4-qF	GAGGGGCCGCCTAGAGATT
FGFR4-qR	CAGGACGATCATGGAGCCT

FGFR1-KD-1F	CCGGCAGAGGAGAAAGAAACAGATACTCGAGTATCTGTTTCTTTCTCCTCTGTTTTTG
FGFR1-KD-1R	AATTCAAAAACAGAGGAGAAAGAAACAGATACTCGAGTATCTGTTTCTTTCTCCTCTG
FGFR1-KD-2F	CCGGCCCTCCCAGATGTTGGACCAACTCGAGTTGGTCCAACATCTGGGAGGGTTTTTG
FGFR1-KD-2R	AATTCAAAAACCCTCCCAGATGTTGGACCAACTCGAGTTGGTCCAACATCTGGGAGGG
FGFR4-KD-1F	CCGGGCTGGAGAGCTGCTATGCTAACTCGAGTTAGCATAGCAGCTCTCCAGCTTTTTG
FGFR4-KD-1R	AATTCAAAAAGCTGGAGAGCTGCTATGCTAACTCGAGTTAGCATAGCAGCTCTCCAGC
FGFR4-KD-2F	CCGGGCCGACACAAGAACATCATCACTCGAGTGATGATGTTCTTGTGTCGGCTTTTTG
FGFR4-KD-2R	AATTCAAAAAGCCGACACAAGAACATCATCACTCGAGTGATGATGTTCTTGTGTCGGC
